# Comprehensive Phylogenetic Analysis of Bovine Non-*aureus* Staphylococci Species Based on Whole-Genome Sequencing

**DOI:** 10.3389/fmicb.2016.01990

**Published:** 2016-12-20

**Authors:** Sohail Naushad, Herman W. Barkema, Christopher Luby, Larissa A. Z. Condas, Diego B. Nobrega, Domonique A. Carson, Jeroen De Buck

**Affiliations:** ^1^Department of Production Animal Health, Faculty of Veterinary Medicine, University of CalgaryCalgary, AB, Canada; ^2^Canadian Bovine Mastitis and Milk Quality Research NetworkSt-Hyacinthe, QC, Canada; ^3^Department of Large Animal Clinical Sciences, Western College of Veterinary Medicine, University of SaskatchewanSaskatoon, SK, Canada

**Keywords:** Non-*aureus* staphylococci, coagulase-negative staphylococci, bovine intramammary infection, phylogenetic trees, whole-genome sequencing

## Abstract

Non-*aureus* staphylococci (NAS), a heterogeneous group of a large number of species and subspecies, are the most frequently isolated pathogens from intramammary infections in dairy cattle. Phylogenetic relationships among bovine NAS species are controversial and have mostly been determined based on single-gene trees. Herein, we analyzed phylogeny of bovine NAS species using whole-genome sequencing (WGS) of 441 distinct isolates. In addition, evolutionary relationships among bovine NAS were estimated from multilocus data of 16S rRNA, *hsp60, rpoB, sodA*, and *tuf* genes and sequences from these and numerous other single genes/proteins. All phylogenies were created with FastTree, Maximum-Likelihood, Maximum-Parsimony, and Neighbor-Joining methods. Regardless of methodology, WGS-trees clearly separated bovine NAS species into five monophyletic coherent clades. Furthermore, there were consistent interspecies relationships within clades in all WGS phylogenetic reconstructions. Except for the Maximum-Parsimony tree, multilocus data analysis similarly produced five clades. There were large variations in determining clades and interspecies relationships in single gene/protein trees, under different methods of tree constructions, highlighting limitations of using single genes for determining bovine NAS phylogeny. However, based on WGS data, we established a robust phylogeny of bovine NAS species, unaffected by method or model of evolutionary reconstructions. Therefore, it is now possible to determine associations between phylogeny and many biological traits, such as virulence, antimicrobial resistance, environmental niche, geographical distribution, and host specificity.

## Introduction

The genus *Staphylococcus* currently consists of 52 species and 28 subspecies. Phylogeny and classification of this genus is under active investigation and has been subject to substantial revisions (Kloos et al., 1998a,b; Svec et al., 2004; Taponen et al., 2012). The non-*aureus* staphylococci (NAS), previously referred to as coagulase-negative staphylococci [although some species have a variable response to the coagulase test (Dos Santos et al., 2016)], are the most frequently isolated microorganisms from the mammary gland of dairy cows and increasingly recognized as etiologic agents of intramammary infection (IMI) in cattle worldwide (Pyörälä and Taponen, 2009; Sampimon et al., 2009; Thorberg et al., 2009; De Vliegher et al., 2012). Although the NAS are primarily associated with subclinical or mild clinical mastitis (Honkanen-Buzalski et al., 1994; Supré et al., 2011), IMI with NAS decrease quality and quantity of milk produced and can moderately increase somatic cell count (Schukken et al., 2009; Taponen and Pyörälä, 2009; De Vliegher et al., 2012; Condas et al., in review). Some species of NAS are capable of persisting in the udder for months or even throughout the lactation (Aarestrup et al., 1995; Laevens et al., 1997; Taponen et al., 2007; Thorberg et al., 2009). Additionally, NAS contain important virulence factors (Zhang et al., 2003; Otto, 2013; Vanderhaeghen et al., 2014), have a high level of antimicrobial resistance (Rajala-Schultz et al., 2009; Frey et al., 2013; Taponen et al., 2015), and can cause chronic IMI (Gillespie et al., 2009; De Vliegher et al., 2012). In contrast, NAS have also been reported to have a protective role against major IMI-related pathogens (Matthews et al., 1990; De Vliegher et al., 2004). Contradictory findings among studies regarding pathogenicity of NAS is potentially related to classifying disparate species and strains of staphylococci as one group (Woodward et al., 1987, 1988; Matthews et al., 1990; Piepers et al., 2009; Vanderhaeghen et al., 2014). However, NAS are a heterogeneous group of numerous species and it is, therefore, expected that individual NAS species will have different effects on udder health and production (Piepers et al., 2009; Vanderhaeghen et al., 2014). To determine effects of individual or closely related species on udder health and to investigate whether these variable effects align with their phylogenetic relationship, a complete understanding of species relatedness in the NAS group is essential.

Early approaches to understanding staphylococcal relationships and phylogenies were primarily based on sequence analysis of the 16S rRNA gene and other genes, such as *rpoB* (β-subunit of RNA polymerase), *tuf* (elongation factor Tu), cpn60 (heat shock protein 60), and *dnaJ* (heat shock protein 40) (Takahashi et al., 1999; Kwok and Chow, 2003; Shah et al., 2007; Ghebremedhin et al., 2008; Lamers et al., 2012). However, phylogenies based on these single genes displayed contradictory topologies when compared to each other (Ghebremedhin et al., 2008; Lamers et al., 2012). In contrast, utilization of phylogenies based on larger datasets of sequences from multiple genes provides significantly greater resolution of phylogenetic relationships between organisms (Rokas et al., 2003; Wu et al., 2009; Naushad et al., 2015a,b). For example, a recent multi-gene phylogeny of *Staphylococcus* spp., based on the 16S rRNA, *dnaJ, rpoB*, and *tuf* genes (Lamers et al., 2012), provided clear and robust support for many interspecies relationships among recently diverged NAS species. Notwithstanding, for more ancient lineages, there were several conflicting phylogenies among various gene trees.

In this study, we completed whole genome sequencing (WGS) of 441 bovine NAS isolates and utilized WGS data to construct robust and well-resolved phylogenetic trees based on all genes in the core genome of the NAS, thereby providing a highly reliable foundation for understanding interspecies relatedness among the NAS. We also produced several housekeeping (ribosomal and information transfer) gene-based phylogenetic trees to investigate the reliability of specific single gene trees and to determine the gene or group of genes which most closely approximate the phylogeny produced by the core genome. Lastly, we investigated effects of various phylogenetic reconstruction methods on NAS phylogenies in order to determine effects of various parameters and models of evolution on the final topology of NAS phylogenies.

## Materials and methods

### Selection of isolates

A total of 441 NAS isolates (Table 1) were selected for WGS from 5507 isolates obtained from bovine staphylococcal IMI stored at the Mastitis Pathogen Collection of the Canadian Bovine Mastitis and Milk Quality Research Network (CBMQRN). Selection was randomized over the 25 NAS species previously identified (Mellmann et al., 2006; Condas et al., in review) which originated from 87 herds distributed over four geographic regions encompassing Atlantic Canada (Nova Scotia, Prince Edward Island, New Brunswick), Central Canada (Québec and Ontario), and Western Canada (Alberta) (Reyher et al., 2011). Inclusion criteria of isolates were: (1) inclusion of 68 NAS isolated from clinical mastitis samples; (2) inclusion of 26 NAS isolates with multi-drug resistance (MDR); (3) for uncommon species (defined as <20 unique isolations at cow level), inclusion of one arbitrarily selected isolate of that species per cow; (4) one isolate per cow for all other species, until achieving the final number. Our criteria allowed two isolates of the same NAS species isolated from the same cow to be included (i.e., *S. chromogenes* isolated from clinical mastitis and subclinical mastitis with MDR in the same cow), although the majority of isolates originated from different cows (Table 1).

**Table 1:** **Distribution of NAS species that were selected for WGS, obtained from Canadian Bovine Mastitis and Milk Quality Research Network**.

**Species name**	**Total isolates sequenced**	**Alberta**			**Ontario**			**Québec**			Atlantic Canada^*^		
		**N**	**Herds**	**Cows**	**N**	**Herds**	**Cows**	**N**	**Herds**	**Cows**	**N**	**Herds**	**Cows**
*S. agnetis*	13	3	1	2	5	4	4	3	3	3	2	2	2
*S. arlettae*	15	3	2	3	9	9	9	3	3	3	0	0	0
*S. auricularis*	2	1	1	1	1	1	1	0	0	0	0	0	0
*S. capitis*	22	0	0	0	10	8	10	6	5	6	6	6	6
*S. caprae*	1	0	0	0	1	1	1	0	0	0	0	0	0
*S. chromogenes*	83	30	12	27	29	17	26	6	5	6	18	11	17
*S. cohnii*	24	2	2	2	12	9	12	8	6	7	2	2	2
*S. devriesei*	8	3	2	3	3	3	3	0	0	0	2	2	2
*S. epidermidis*	26	5	2	5	8	8	8	7	6	7	6	5	6
*S. equorum*	17	8	6	8	5	5	5	3	2	3	1	1	1
*S. fleurettii*	2	2	1	2	0	0	0	0	0	0	0	0	0
*S. gallinarum*	21	3	3	3	8	7	7	7	5	5	3	3	3
*S. haemolyticus*	29	9	5	9	8	8	8	5	5	5	7	6	7
*S. hominis*	11	0	0	0	2	2	2	7	5	7	2	2	2
*S. hyicus*	3	0	0	0	0	0	0	2	2	2	1	1	1
*S. kloosii*	1	0	0	0	1	1	1	0	0	0	0	0	0
*S. nepalensis*	2	0	0	0	2	2	2	0	0	0	0	0	0
*S. pasteuri*	6	0	0	0	0	0	0	6	4	6	0	0	0
*S. saprophyticus*	16	0	0	0	5	5	5	8	6	8	3	3	3
*S. sciuri*	29	8	6	8	9	9	9	8	7	7	4	3	4
*S. simulans*	42	6	5	6	19	13	18	13	10	13	4	4	4
*S. succinus*	15	3	3	3	5	4	5	1	1	1	6	5	6
*S. vitulinus*	6	2	2	2	1	1	1	0	0	0	3	3	3
*S. warneri*	19	2	2	2	8	6	7	5	2	5	4	4	4
*S. xylosus*	28	8	5	8	8	5	8	11	9	11	1	1	1
Total	441	98	60	94	159	128	152	109	86	105	75	64	74

* *Nova Scotia, Prince Edward Island, and New Brunswick*.

### DNA extraction and whole genome sequencing

All isolates obtained were grown on 5% defibrinated sheep blood agar plates (BD Diagnostics, Mississauga, ON, Canada) with subsequent incubation at 35°C for 24 h to yield single colonies. For each of these isolates, colonies were picked from blood agar plates and suspended in Bacto Brain Heart Infusion broth (BD Diagnostics), and incubated at 37°C overnight. Genomic DNA for all isolates was extracted with DNeasy Blood and Tissue Kit (Qiagen, Toronto, ON, Canada), according to the corresponding protocol for Gram-positive bacteria. Quality and quantity of DNA was checked using a NanoVue plus spectrophotometer (GE Healthcare Life Sciences, Mississauga, ON, Canada) and the Qubit 2.0 fluorometer (Invitrogen, Burlington, ON, Canada). Each DNA sample was diluted to a final concentration of 0.2 ng/μl. Sequencing of these samples was performed using the Illumina MiSeq platform (Illumina, San Diego, CA, USA); DNA libraries for sequencing were prepared using a Nextera XT DNA library preparation kit (Illumina, San Diego, CA, US). All sequencing steps, including cluster generation, paired-end sequencing (2 × 250 bp), and primary data analysis for quality control, were performed on the instrument.

### Genome assembly and annotation

Sequence reads obtained from the MiSeq platform were checked for poorly sequenced regions and Illumina adapters sequences were trimmed using cutadapt (Martin, 2011), implemented in Trim Galore! 0.4.0 (with default parameters). After trimming sequences, filtered reads were assembled into contigs using the *de novo* assembly program SPAdes version 3.6.0 (Nurk et al., 2013), employing built-in error correction and default parameters. To determine average coverage of sequencing, reads were mapped back to the assembled genome using BWA 0.7.12-r1039 (Li and Durbin, 2010) and depth of sequencing for each contig was plotted using BEDTools (Quinlan and Hall, 2010). Genome annotations and gene predictions for contigs larger than 200 bp were performed with Prokka 1.11 (Seemann, 2014), using the provided *Staphylococcus* database. Quality of the assembled genomes and assembly metrics was determined using Quast (Gurevich et al., 2013). The entire genome assembly process was automated using the Snakemake workflow engine (Köster and Rahmann, 2012). The data was submitted to NCBI under BioProject ID PRJNA342349.

### Single protein based phylogenetic analysis

Phylogenetic analyses were performed on 24 highly conserved housekeeping proteins (Supplementary Table 1). Sequences for each of these 24 proteins for all NAS species were retrieved from the genomes using BLAST+2.2.31 (Camacho et al., 2009). The sequence for *Macrococcus caseolyticus*, an outgroup species, was downloaded from NCBIs GenBank database. Multiple sequence alignments for these proteins were created using MUSCLE v3.8.31 (Edgar, 2004). The resulting alignments were used for phylogenetic analysis. Phylogenetic trees, based on 100 bootstrap replicates, were constructed by employing Maximum-Likelihood (ML), Maximum-Parsimony (MP), and Neighbor-Joining (NJ) methods using MEGA 6.0 (Tamura et al., 2013). Evolutionary distances for ML and NJ methods were computed using a JTT matrix-based model (Jones et al., 1992). Maximum-Parsimony trees were obtained using the Subtree-Pruning-Regrafting (SPR) algorithm (Nei and Kumar, 2000).

### Single gene/s and multilocus sequence analysis

Phylogenetic trees were constructed based on full-length sequences of 16S rRNA, *hsp60, rpoB, sodA*, and *tuf* genes. Full-length sequences of these genes for NAS species were obtained using BLAST+2.2.31 (Camacho et al., 2009). Multiple sequence alignments for each of gene were created using MUSCLE v3.8.31 (Edgar, 2004). Maximum-Likelihood, MP and NJ trees based on these sequence alignments were created using 100 bootstrap replicates in MEGA 6.0 (Tamura et al., 2013).

Multilocus sequence analysis was performed on nucleotide sequences of the 16S rRNA, *hsp60, rpoB, sodA*, and *tuf* genes. Individual gene alignments were manually concatenated to create a combined dataset of these five genes. Poorly aligned regions from this concatenated alignment were removed using Gblocks 0.92 (Castresana, 2000) with default settings except the allowed gap position parameter which was changed to 0.5 (50%). Maximum-Likelihood, MP and NJ trees based on 100 bootstrap replicates of this dataset were constructed using MEGA 6.0 (Tamura et al., 2013). For all trees, evolutionary distances for ML, MP, and NJ methods were computed using the General Time Reversible model (Nei and Kumar, 2000), Subtree-Pruning-Regrafting (Nei and Kumar, 2000), and Kimura 2-parameter model (Kimura, 1980), respectively. Codon positions included were 1st+2nd+3rd+Noncoding. All positions containing gaps and missing data were eliminated from final alignments. All trees were rooted using *Macrococcus caseolyticus*, and gene sequences for this species were downloaded from NCBI GenBank database.

### NAS phylogenetic tree—PhyloPhlAn

A genome-based phylogenetic tree of 441 NAS isolates was constructed using the published pipeline PhyloPhlAn (Segata et al., 2013). Briefly, the PhyloPhlAn approach is based on the use of 400 ubiquitous and phylogenetically informative proteins. Orthologs of these proteins in NAS genomes were detected using USEARCH v5.2.32 (Edgar, 2010). Multiple sequence alignments of these proteins were generated using MUSCLE v3.8.31 (Edgar, 2004). A final concatenated dataset containing 4231 aligned amino acid positions was generated. Final tree construction was performed using FastTree version 2.1 (Price et al., 2010). To determine effects of various evolutionary models on the resulting tree, Maximum-Likelihood, MP, and NJ trees based on 100 bootstrap replicates of the final alignment were also constructed using MEGA 6.0 (Tamura et al., 2013). For ML and NJ trees, evolutionary distances were computed using a JTT matrix-based model (Jones et al., 1992), whereas the Subtree-Pruning-Regrafting algorithm (Nei and Kumar, 2000) was used for MP tree construction.

### NAS core genome phylogenetic tree

A phylogenetic tree of all NAS isolates, rooted using *Macrococcus caseolyticus*, was created based on the core genome of the bovine NAS group. The core set of NAS proteins were identified using the UCLUST algorithm (Edgar, 2010). Protein families with at least 30% sequence identity and 50% sequence length in 441 NAS isolates were considered core. However, protein families present in <80% of the input genomes were excluded from further analysis. Proteins families which contained potential paralogous sequences (duplicated sequence in same genome) were also excluded from further analysis. Each protein family was individually aligned using MAFFT 7 (Katoh and Standley, 2013). Aligned amino acid positions which contained gaps in more than 50% of genomes, were excluded from further analysis. Remaining amino acid positions were concatenated to create a combined dataset consisting of 128,080 aligned amino acids. A maximum-likelihood tree based on this alignment was constructed using FastTree 2.1 (Price et al., 2010), using the Whelan and Goldman substitution model (Whelan and Goldman, 2001) and JTT matrix-based model (Jones et al., 1992).

### Comparison of phylogenetic trees

Genome-based phylogenetic trees (PhyloPhlAn, ML, MP, NJ) and Core-Genome-Tree (CGT) were visually inspected and compared. Single gene and protein trees were compared with each other and with CGT. Topological differences among them were computed using Robinson-Foulds (RF) distance matrix (Robinson and Foulds, 1981; Makarenkov and Leclerc, 2000), implemented as webserver; T-REX (Tree and reticulogram REConstruction, www.trex.uqam.ca; Boc et al., 2012). To facilitate comparisons of our results, RF distance scores were normalized using the maximum possible distance between two trees, calculated according to 2(N-3) where N represents the total number of taxa on a tree (Kupczok et al., 2010; Bernard et al., 2016). We designated these as normalized-Robinson-Foulds (nRF) scores, with values ranging from 0 (0%) to 1 (100%), and nRF = 0 indicating that topologies of two trees under investigation are congruent. Consequently, higher nRF score indicate a low level of congruence (or high level of incongruence) between two tree topologies.

## Results

### Whole-genome based phylogenetic analysis of NAS

**Figure 1:**
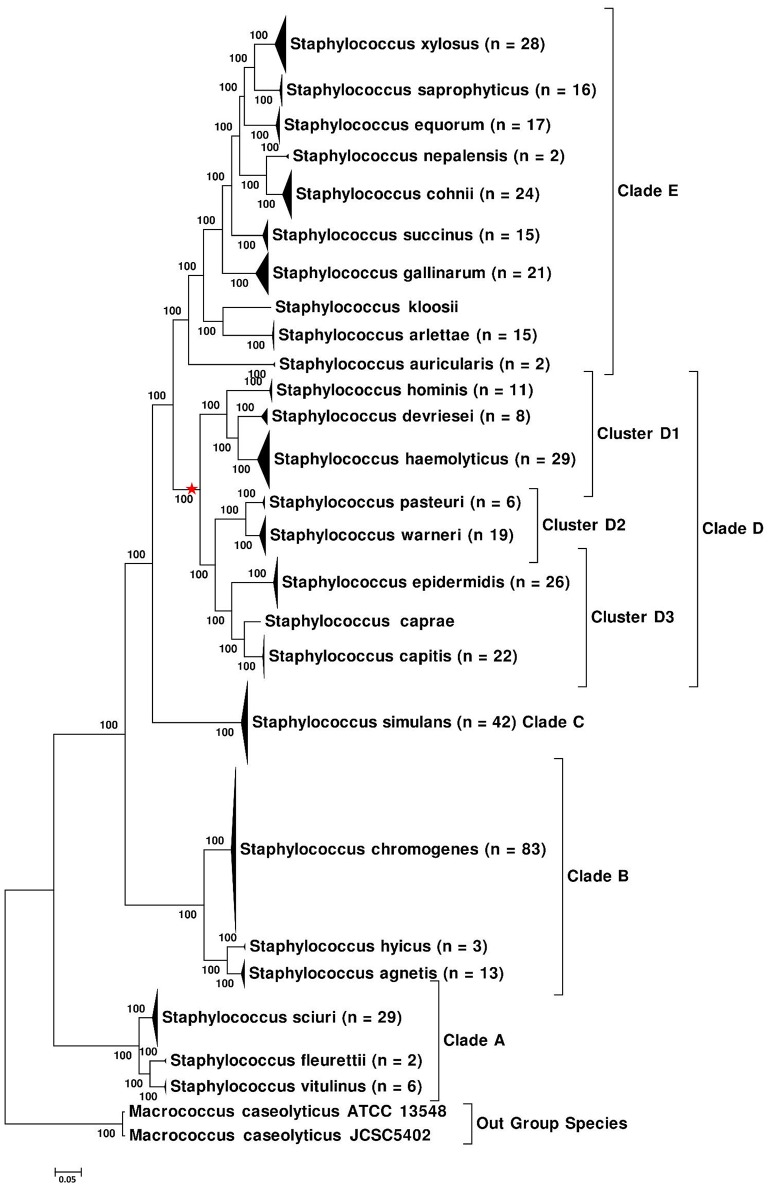
**Core-Genome-Tree of NAS, showing the branching of NAS species into five distinct clades**. Multiple isolates of the same species were collapsed and total number of isolates for each species are shown after the species name. The evolutionary history of NAS was inferred using the Maximum Likelihood method based on the Whelan and Goldman substitution model (Whelan and Goldman, 2001) and JTT model (Jones et al., 1992), resulting in identical topology for both models. The percentage of trees in which the associated taxa clustered together is shown next to the branches. The tree is drawn to scale, with branch lengths measured in number of substitutions per site. Red star on the tree indicates phylogenetic placement of *S. aureus*.

Five phylogenetic trees were constructed based on WGS data of NAS isolates. The first of these trees was constructed using a core genome dataset of NAS species (Figure 1), whereas the second tree (Supplementary Figure 1) was constructed using PhyloPhlAn (Segata et al., 2013). The same dataset from aligned sequences, obtained from PhyloPhlAn was also used to construct the ML (Supplementary Figure 2), MP, (Supplementary Figure 3), and NJ trees (Supplementary Figure 4). Within these trees, species of the NAS group branched into five distinct monophyletic and statistically well-supported clades (100% nodal support) in all phylogenetic reconstructions. We referred to them as Clade A, Clade B, Clade C, Clade D, and Clade E, which contained 3, 3, 1, 8, and 10 NAS species, respectively (Figure 1). The phylogeny of NAS species was also constructed by excluding 26 multidrug resistant isolates, as they could have introduced selection bias. However, no differences in phylogeny of the NAS species were seen (data not shown).

In all phylogenetic trees, Clade A branched as first or as the most ancient divergence of NAS group and was composed of three NAS species: *S*. *sciuri, S. fleurettii*, and *S. vitulinus*. Within this clade, *S. sciuri* formed a distant cluster basal to *S. fleurettii* and *S. vitulinus*, which appeared as sister taxa to each other (Figure 1 and Supplementary Figures 1–4). The second or Clade B of NAS group contained *S. chromogenes, S. hyicus*, and *S. agnetis*, with *S. hyicus* appearing as sister taxon to *S. agnetis* and *S. chromogenes* forming the basal group (Figure 1 and Supplementary Figures 1–3). However, there was a discrepancy in branching order among these species in the phylogenetic tree constructed using NJ method (Supplementary Figure 4), which placed *S. chromogenes* as sister taxon to *S. hyicus* and displayed *S. agnetis* branching separately. The next lineage of NAS to diverge was constituted by all isolates of a single species *S. simulans* (Clade C). All 42 sequenced isolates of this species diverged independently from other NAS members by a long branch, forming a strongly supported clade (Figure 1 and Supplementary Figures 1–4).

Clade D contained eight species comprised of *S. hominis, S. devriesei, S. haemolyticus, S. pasteuri, S. warneri, S. epidermidis, S. caprae*, and *S. capitis* (Figure 1 and Supplementary Figures 1–4). *Staphylococcus aureus*, a major pathogen of IMI, also shared common ancestor with species of Clade D. Phylogenetic placement of *S. aureus* is indicated on the tree (Figure 1). The eight NAS species of this clade were split into three clusters. The first cluster (D1) was composed of *S. hominis, S. devriesei*, and *S. haemolyticus*. Within this cluster, *S. hominis* appeared as a deeper branching species, whereas *S. devriesei* and *S. haemolyticus* branched together, representing divergence from a common ancestor (Figure 1 and Supplementary Figures 1–4). The second cluster (D2) was formed by two NAS species, *S. pasteuri* and *S. warneri*, grouping together with 100% support in all genomic phylogenies (Figure 1 and Supplementary Figures 1–4). Cluster D3 displayed a closer relationship of *S. caprae* to *S. capitis*, whereas *S. epidermidis* appeared as the basal lineage of this cluster. Monophyletic branching of these clusters within Clade D was supported by 100% bootstrap scores in all trees. The branching order and phylogenetic placement of these cluster groups was the same in all phylogenies (Figure 1 and Supplementary Figures 1–4).

The fifth clade or Clade E was the most recently diverged clade of NAS and contained 10 species, comprised of *S. auricularis, S. kloosii, S. arlettae, S. gallinarum, S. succinus, S. nepalensis, S. cohnii, S. equorum, S. saprophyticus*, and *S. xylosus* (Figure 1 and Supplementary Figures 1–4). Within this clade, *S. auricularis* was the deepest branching species, followed by a cluster group shared by *S. kloosii* and *S. arlettae*. Next, *S. gallinarum* and *S. succinus* diverged as two well-supported separate lineages (Figure 1 and Supplementary Figures 1–4). Within the remaining species, there was a closer relationship of *S. nepalensis* to *S. cohnii* and *S. saprophyticus* to *S. xylosus* (Figure 1 and Supplementary Figures 1–4). All isolates of *S. equorum* branched together as sister taxa to the *S. xylosus*—*S. saprophyticus* group.

### Single gene/s and multilocus sequence analysis

**Figure 2:**
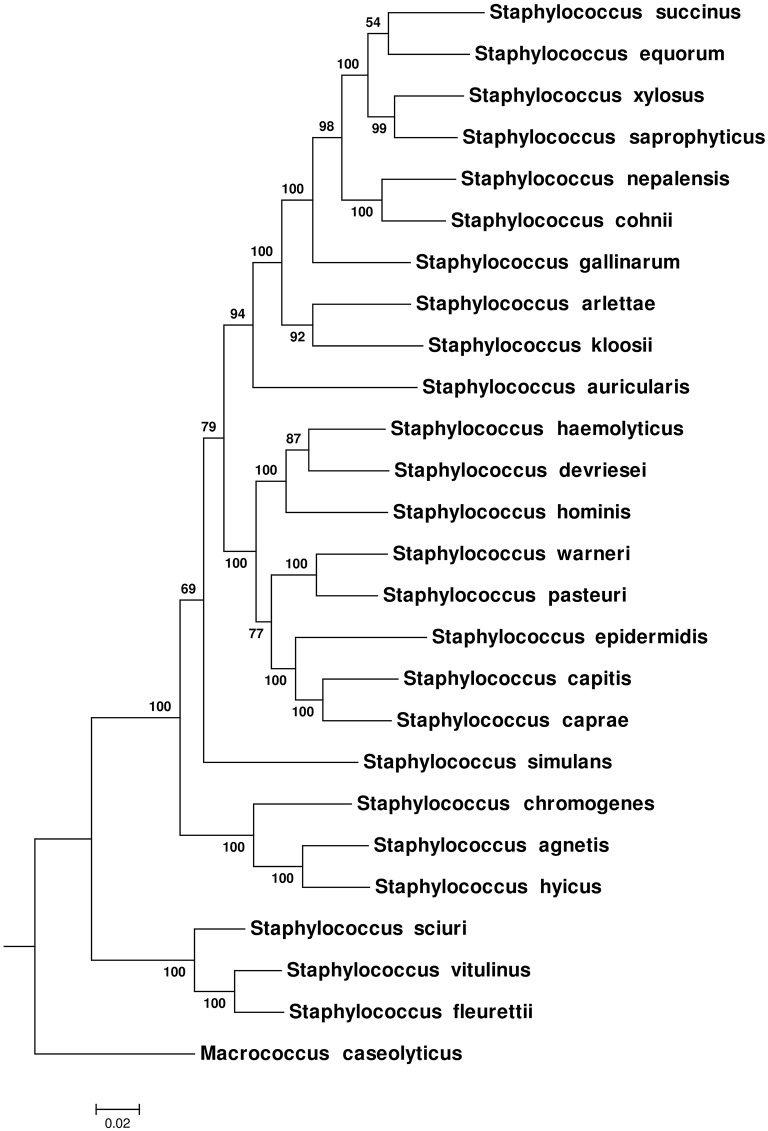
**Maximum-Likelihood phylogenetic tree obtained from multilocus data set of 16S rRNA, ***hsp60***, ***rpoB, sodA***, and ***tuf*** genes**. The tree was constructed using a General Time Reversible model (Nei and Kumar, 2000). Bootstrap scores for each node are presented next to the branches. The tree was drawn to scale, with branch lengths measured in number of substitutions per site.

Phylogenetic relationships among NAS species were also inferred using 16S rRNA, *hsp60, rpoB, sodA*, and *tuf* gene sequences separately and as a multilocus data set. The ML, MP, and NJ trees produced from these genes were compared with each other and with the CGT. The nRF distance-based comparisons among ML, NJ single trees and CGT (Tables 2A,B) and single genes MP trees and CGT (Table 2C) are presented in Table 2. Consistent with the CGT, multilocus ML (Figure 2) and NJ (Supplementary Figure 5) trees displayed branching of NAS species into five distinct clades (Clade A, Clade B, Clade C, Clade D, and Clade E). The MP-multilocus tree (Supplementary Figure 6), however, had a different pattern of clade divergence, and displayed branching of *S. simulans* with *S. auricularis* within Clade E. Hence, Clade C was absent from MP-based phylogeny. Except for the multilocus MP tree, species composition within each clade was identical to the CGT. However, branching order within Clade D and Clade E differed among each other and with CGT. Within Clade D, in the multilocus MP tree, the cluster group containing *S. pasteuri* and *S. warneri* suggested a deeper branching cluster compared to other species of this clade (Supplementary Figure 6). The notable difference within clade E was branching of *S. succinus* and *S. equorum* as sister taxa in all multilocus trees (Figure 2 and Supplementary Figures 5, 6).

**Table 2:** **Comparisons of normalized-Robinson-Foulds (nRF) scores among CGT and single gene ML (A), single gene NJ (B), and single gene MP (C) trees**.

	**CGT**	**Multilocus**	***16S rRNA***	***hsp60***	***rpoB***	***sodA***	***tuf***
**(A)**							
CGT	0^*^						
Multilocus	0.1	0					
*16S rRNA*	0.3	0.3	0				
*hsp60*	0.3	0.3	0.3	0			
*rpoB*	0.1	0.1	0.3	0.3	0		
*sodA*	0.3	0.3	0.4	0.5	0.3	0	
*tuf*	0.3	0.3	0.5	0.4	0.3	0.5	0
**(B)**							
CGT	0^*^						
Multilocus	0.1	0					
*16S rRNA*	0.4	0.4	0				
*hsp60*	0.3	0.3	0.4	0			
*rpoB*	0.1	0.1	0.4	0.3	0		
*sodA*	0.3	0.3	0.3	0.5	0.3	0	
*tuf*	0.3	0.3	0.5	0.4	0.3	0.5	0
**(C)**							
CGT	0^*^						
Multilocus	0.2	0					
*16S rRNA*	0.5	0.5	0				
*hsp60*	0.6	0.5	0.7	0			
*rpoB*	0.3	0.3	0.5	0.5	0		
*sodA*	0.3	0.3	0.6	0.6	0.4	0	
*tuf*	0.3	0.3	0.7	0.5	0.5	0.4	0

* *The colors of the cells represent heat map generated from the nRF scores. nRF value zero (dark green) indicates that the two trees are topologically congruent. Higher nRF scores (dark red) indicate a lower level of congruence between two tree topologies*.

**Figure 3:**
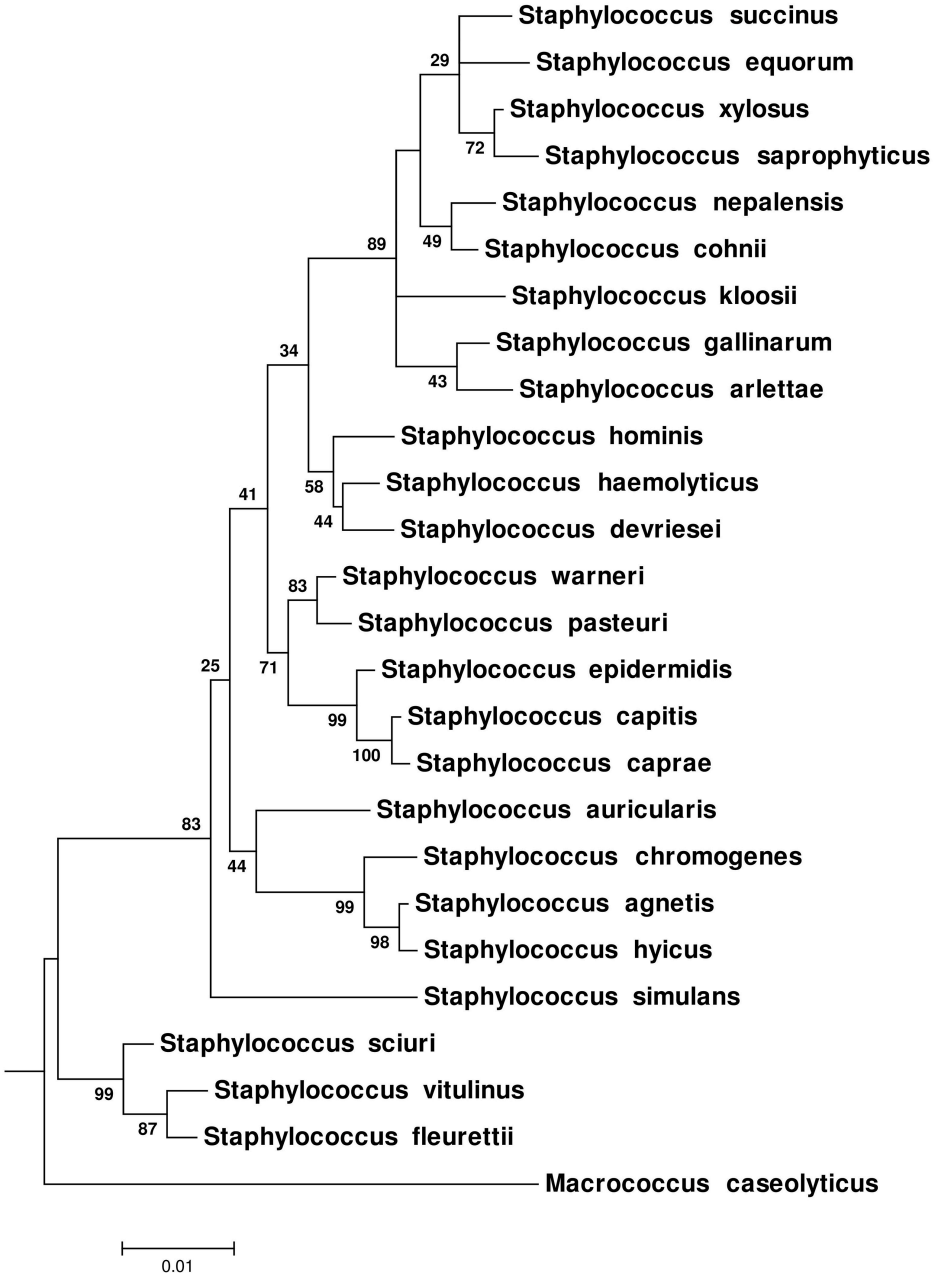
**Maximum-Likelihood phylogenetic tree based on 16S rRNA gene sequences of NAS species**. The tree was constructed using General Time Reversible model (Nei and Kumar, 2000). The tree was drawn to scale, with branch lengths measured in the number of substitutions per site. The values on the nodes represent bootstrap support for each relationship.

Phylogenetic trees (ML, MP, and NJ) based upon 16S rRNA gene (Figure 3 and Supplementary Figures 7, 8), *hsp60* (Supplementary Figures 9–11), *rpoB* (Supplementary Figures 12–14), *sodA* (Supplementary Figures 15–17), and *tuf* (Supplementary Figures 18–20) gene sequences were constructed. The nRF distance-based analyses for ML trees (Table 2A), NJ trees (Table 2B), and MP trees (Table 2C) based upon these five genes, indicated that these trees differed from each other and from the CGT (Table 2). Within the 16S rRNA gene based phylogenies, the only reliably identified relationship was the composition and branching order of *S. sciuri, S. fleurettii*, and *S. vitulinus* within Clade A (Figure 3 and Supplementary Figures 7, 8). All other species had varying patterns of evolutionary placement. Although *S. simulans* branched distinctly in ML and NJ trees, the order of its divergence was different compared to the CGT. In these cases, *S. simulans* branched after divergence of Clade A from the rest of the NAS species (Figure 3 and Supplementary Figure 8), as opposed to branching after separation of Clades A and B in the CGT (Figure 1 and Supplementary Figures 1–4). The branching of S. *simulans* after Clade A had 83 and 80% statistical support in ML (Figure 3) and NJ (Supplementary Figure 8) trees, respectively. In the 16S-rRNA-MP tree, *S. simulans* clustered with other NAS species (Supplementary Figure 7). Within Clade B, *S. auricularis* branched with *S. chromogenes, S. agnetis*, and *S. hyicus* (Figure 3). However, Clade D was not recovered as a monophyletic lineage. The cluster group containing *S. hominis, S. haemolyticus*, and *S. devriesei* appeared as separate taxa distinct from the rest of Clade D species (Figure 3). Except for *S. auricularis*, which clustered with clade B, species composition was invariable within Clade E. However, there was discordance among branching of these species. Additionally, phylogenetic positioning of *S. kloosii* and *S. equorum* was not resolved properly within Clade E (Figure 3).

Phylogenies constructed using *hsp60* gene sequence differed significantly from each other, both in species composition and their hierarchy of branching pattern. For three *hsp60* trees (ML, MP, and NJ), Clade A branches as monophyletic group with 100% bootstrap scores (Supplementary Figures 9–11). However, species of other clades were intermixed. The ML and NJ phylogenetic trees constructed using *rpoB* gene sequence revealed the same major clades. However, in both trees, *S. simulans* branched with *S. chromogenes, S. agnetis*, and *S. hyicus*, species of Clade B. Within Clade D, in both the ML and NJ trees, *S. pasteuri* and *S. warneri* formed a basal branching group compared to other Clade D species. Within Clade E, there were similar branching patterns among ML and NJ trees; the only difference was branching of *S. succinus* after the *S. cohnii—S. nepalensis* pair, as opposed to its divergence after *S. gallinarum* in CGT. Except for Clade A, the MP tree (Supplementary Figure 13) based on the *rpoB* gene, had a largely different pattern of evolutionary relationships among NAS species. Phylogenetic trees based on the *sodA* gene only revealed formation of Clades A and B, similar to CGT (Supplementary Figures 15–17), whereas remaining species of other clades (Clade C, D, and E) had a polyphyletic grouping pattern. For ML and NJ method-based phylogenies obtained from *tuf* gene, NAS species branched into the same five distinct clades as the CGT (Supplementary Figures 18, 20). However, in the MP (*tuf*) tree, *S. simulans* grouped with species of Clade D (Supplementary Figure 19). Hierarchy and clustering patterns among members of Clade D and Clade E differed among various phylogenies, based on the *tuf* gene.

### Single protein/s (translated genes) based phylogeny of NAS

Evolutionary relationships of NAS species were also investigated, based on 24 housekeeping protein sequences (Supplementary Table 1). Phylogenies obtained from each protein sequence were examined by various methods of evolutionary estimations (ML, MP, and NJ). Three trees (ML, MP, and NJ) produced from each single protein, were compared with each other and with CGT. The nRF distances between each of ML, NJ, and MP single protein trees and CGT are presented (Tables 3–5, respectively). Differences and similarities in species composition and branching order among species of various clades among trees are presented (Table 6).

**Table 3:**
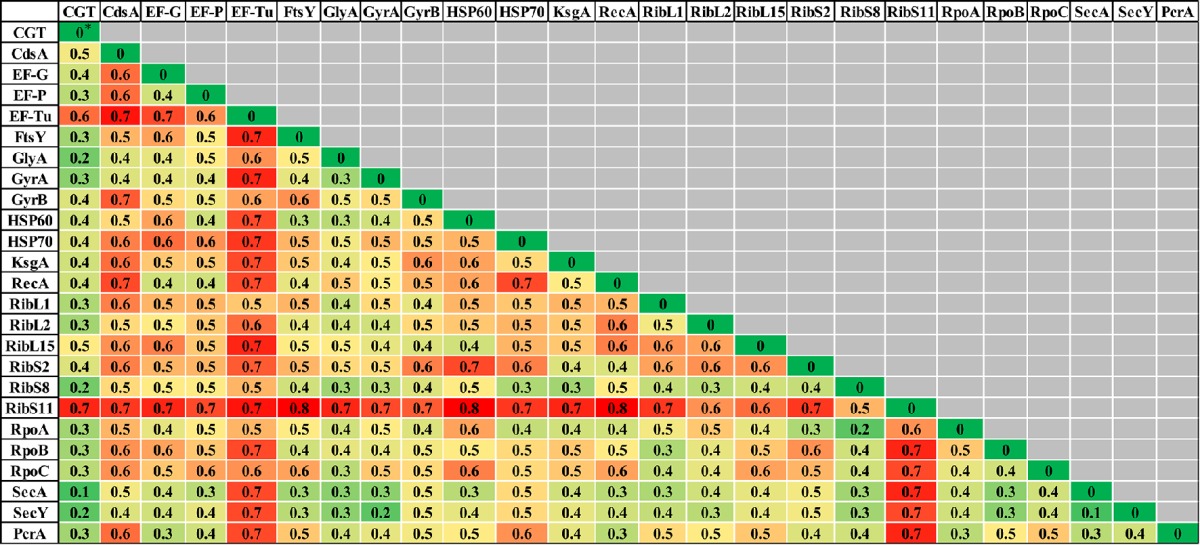
**Normalized-Robinson-Foulds distance score comparisons of CGT and single proteins ML trees**.

^*^The colors of the cells represent heat map generated from the nRF scores. nRF value zero (dark green) indicates that the two trees are topologically congruent. Higher nRF scores (dark red) indicate a lower level of congruence between two tree topologies.

**Table 4:**
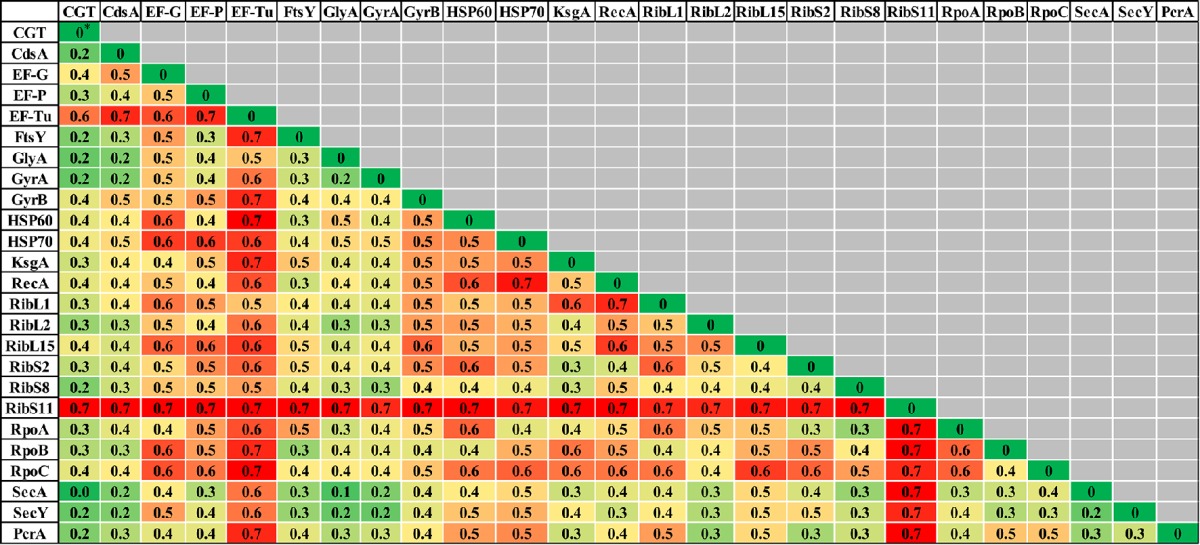
**Normalized-Robinson-Foulds distance score comparisons of CGT with single protein NJ trees**.

^*^The colors of the cells represent heat map generated from the nRF scores. nRF value zero (dark green) indicates that the two trees are topologically congruent. Higher nRF scores (dark red) indicate a lower level of congruence between two tree topologies.

**Table 5:**
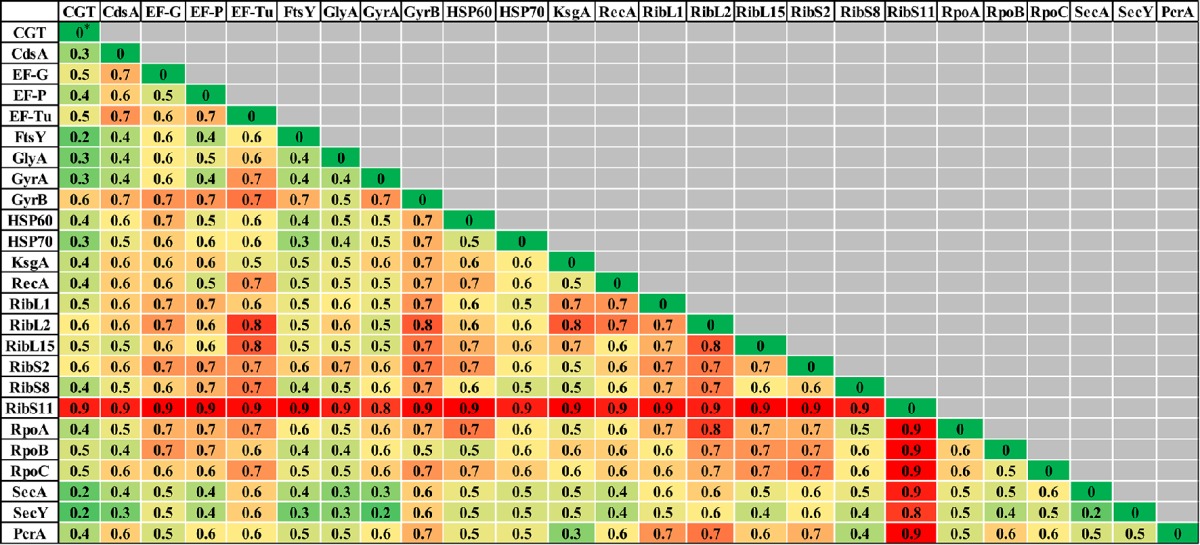
**Normalized-Robinson-Foulds distance score comparisons of CGT with single protein MP trees**.

^*^The colors of the cells represent heat map generated from the nRF scores. nRF value zero (dark green) indicates that the two trees are topologically congruent. Higher nRF scores (dark red) indicate a lower level of congruence between two tree topologies.

**Table 6:** **Description of similarities and divergences among phylogenetic trees of 24 proteins most commonly applied in CNS species identification and phylogenetic estimations**.

**Protein/Method**	**Clade A**						**Clade B**						**Clade C**			**Clade D**						**Clade E**					
	**Species**			**Branching**			**Species**			**Branching**			**Species**			**Species**			**Branching**			**Species**			**Branching**		
	MP^*^	**NJ**	**ML**	**MP**	**NJ**	**ML**	**MP**	**NJ**	**ML**	**MP**	**NJ**	**ML**	**MP**	**NJ**	**ML**	**MP**	**NJ**	**ML**	**MP**	**NJ**	**ML**	**MP**	**NJ**	**ML**	**MP**	**NJ**	**ML**
CdsA	MP^§^	NJ	ML	.^$^	NJ	.	MP	NJ	ML	MP	NJ	.	MP	NJ	ML	.	.	.	.	.	.	MP	NJ	ML	.	.	.
EF-G	MP	NJ	ML	MP	NJ	ML	MP	NJ	ML	MP	NJ	ML	.	.	.	MP	NJ	ML	.	.	.	.	.	.	.	.	.
EF-P	MP	NJ	ML	MP	NJ	ML	MP	NJ	ML	MP	NJ	ML	.	.	.	.	.	.	.	.	.	.	.	.	.	.	.
EF-Tu	.	NJ	ML	.	NJ	.	MP	NJ	ML	MP	NJ	ML	.	.	.	.	.	.	.	.	.	.	.	.	.	.	.
FtsY	MP	NJ	ML	MP	NJ	.	.	NJ	ML	.	NJ	.	.	NJ	ML	MP	NJ	ML	.	.	.	MP	NJ	ML	.	.	.
GlyA	MP	NJ	ML	MP	NJ	ML	MP	NJ	ML	.	NJ	.	.	.	.	.	NJ	.	.	NJ	.	MP	NJ	ML	MP	.	.
GyrA	MP	NJ	ML	MP	NJ	ML	MP	NJ	ML	.	NJ	ML	MP	NJ	ML	MP	NJ	ML	MP	.	.	MP	NJ	ML	.	.	.
GyrB	MP	NJ	ML	MP	NJ	ML	.	NJ	ML	.	NJ	.	.	.	.	MP	NJ	.	.	.	.	.	.	.	.	.	.
HSP60	MP	NJ	ML	MP	NJ	ML	MP	NJ	ML	MP	NJ	.	.	NJ	.	MP	NJ	.	.	.	.	MP	NJ	ML	.	NJ	.
HSP70	MP	NJ	ML	MP	NJ	ML	MP	NJ	ML	MP	NJ	ML	MP	NJ	ML	.	.	.	.	.	.	MP	.	ML	.	.	.
KsgA	MP	NJ	ML	MP	NJ	ML	MP	NJ	ML	MP	NJ	ML	.	.	.	.	NJ	ML	.	.	.	.	.	.	.	.	.
RecA	MP	NJ	ML	MP	.	.	MP	NJ	ML	MP	NJ	ML	.	.	.	MP	NJ	ML	.	.	.	.	.	.	.	.	.
RibL1	MP	NJ	ML	MP	NJ	ML	MP	.	.	MP	.	.	MP	.	.	.	NJ	.	.	.	.	.	NJ	ML	.	.	.
RibL15	MP	NJ	ML	MP	NJ	ML	MP	NJ	ML	MP	.	ML	.	NJ	.	.	.	.	.	.	.	.	.	.	.	.	.
RibL2	MP	NJ	ML	MP	NJ	ML	.	NJ	.	.	NJ	.	.	NJ	.	.	NJ	ML	.	.	.	.	NJ	ML	.	.	.
RibS11	.	.	ML	.	.	ML	.	.	.	.	.	.	.	.	.	.	.	.	.	.	.	.	.	.	.	.	.
RibS2	MP	NJ	ML	.	.	.	MP	NJ	.	MP	NJ	.	MP	NJ	.	.	.	.	.	.	.	.	.	.	.	.	.
RibS8	MP	NJ	ML	MP	NJ	ML	MP	NJ	ML	MP	NJ	ML	MP	NJ	ML	MP	.	ML	.	.	.	.	.	ML	.	.	.
RpoA	MP	NJ	ML	.	NJ	.	MP	NJ	ML	MP	NJ	ML	.	.	.	.	.	.	.	.	.	.	.	.	.	.	.
RpoB	MP	NJ	ML	.	NJ	ML	MP	NJ	ML	MP	NJ	ML	MP	NJ	.	MP	NJ	ML	.	.	.	MP	NJ	ML	.	NJ	.
RpoC	MP	NJ	ML	MP	NJ	ML	.	.	ML	.	.	ML	.	.	ML	.	.	.	.	.	.	.	.	.	.	.	.
SecA	MP	NJ	ML	MP	NJ	ML	MP	NJ	ML	MP	NJ	ML	MP	.	.	.	NJ	ML	.	NJ	.	.	NJ	.	.	NJ	.
SecY	MP	NJ	ML	MP	NJ	ML	MP	NJ	ML	MP	NJ	ML	MP	.	ML	MP	NJ	ML	.	.	.	MP	NJ	ML	.	.	.
PcrA	MP	NJ	ML	MP	NJ	ML	MP	NJ	ML	MP	NJ	ML	.	NJ	.	MP	NJ	ML	.	NJ	.	.	.	.	.	.	.

* *Methods of tree construction MP, Maximum parsimony; NJ, Neighbor-joining; ML, Maximum parsimony*.

§ *Color shaded areas in orange, green, blue, yellow, and pink indicates the methods in each the presence of species, and branching at respective clades were in agreement to clades observed in CGT*.

$ *Gray areas indicate disagreement between the species, and branching at respective clade compared to CGT*.

Species composition and branching order for Clade A were retrieved in most phylogenetic reconstructions (Table 3) except MP trees [Elongation factor Tu (EF-Tu) and ribosomal protein S11 (RibS11)] and NJ tree [ribosomal protein S11 (RibS11)] which could not recover Clade A. Among protein trees that depicted branching of Clade A as monophyletic group, there were branching order differences among species of Clade A in MP trees, based upon cytidine diphosphate (CDP) diglyceride synthetase (CdsA), ribosomal protein S2 (RibS2) and RNA polymerase subunits α and β protein sequences (RpoA and RpoB). Discordance in branching of Clade A species was also apparent in NJ trees constructed from RecA, ribosomal protein S2 (RibS2) and ML tree reconstructed from CDP diglyceride synthetase (CdsA), elongation factor Tu (EF-Tu), cell division protein FtsY, RecA, ribosomal protein S2 (RibS2), and RNA polymerase α subunit (RpoA) protein sequences.

Similar to Clade A, most phylogenetic estimation methods produced Clade B as an independent and distinct monophyletic group, except in MP trees built from cell division protein FtsY, gyrase B (GyrB), ribosomal protein L2 (RibL2), RibS11, and RNA polymerase β′ (RpoC) subunit protein sequences. Within Clade B, dissimilarities in branching among its species were seen in MP [serine hydroxymethyl transferase (GlyA) and gyrase A (GyrA)], NJ [ribosomal protein L15 (RibL15)] and ML trees [cell division protein FtsY, serine hydroxymethyl transferase (GlyA), gyrase B (GyrB), and HSP60]. Clade C was considered accurate only when it branched after Clades A and B in phylogenetic trees. Several protein sequences, namely, CDP diglyceride synthetase (CdsA), gyrase A (GryA), HSP70 and ribosomal protein S8 (RibS8), produced precise branching of Clade C in ML, MP, and NJ trees. Accurate branching of Clade C was also recovered in MP trees constructed from ribosomal protein L1 (RibL1) and RibS2, preprotein translocase SecA, SecY, and NJ trees, based on cell division protein FtsY, ribosomal protein L2, L15, and S2 (RibL2, RibL15, and RibS2) and PcrA helicase. ML trees constructed using sequences from cell division protein FtsY and preprotein translocase SecY also produced correct branching of Clade C (identical to CGT).

Species composition for Clade D, matching to CGT, was obtained in ML, MP, and NJ phylogenies constructed using sequences from elongation factor G (EF-G), cell division protein FtsY, Gyrase A (GyrA), RecA, RNA polymerase subunit β (RpoB), preprotein translocase SecY and PcrA helicase. However, identical branching patterns among species of Clade D were only observed in MP (GyrA) and NJ trees constructed from serine hydroxymethyl transferase (GlyA), preprotein translocase SecA and PcrA helicase protein sequences. Accurate species composition within Clade E was recovered in all phylogenetic estimations (ML, MP, and NJ) obtained from CDP diglyceride synthetase (CdsA), cell division protein FtsY, serine hydroxymethyl transferase (GlyA), gyrase A (GyrA), HSP60, RNA polymerase subunit β (RpoB), and preprotein translocase SecY. However, the matching branching order among the species of Clade E to CGT was only present in one MP tree [Serine hydroxymethyl transferase (GlyA)] and three NJ trees [HSP60, RNA polymerase subunit β (RpoB), and preprotein translocase SecA].

## Discussion

The role of NAS species in bovine udder health is controversial. Several studies concluded NAS are the principal cause of IMI (Piepers et al., 2007; Sampimon et al., 2009). In contrast, other researchers reported a protective effect of naturally occurring IMI with NAS (Matthews et al., 1990; De Vliegher et al., 2004). Species-specific studies aiming to determine the role of individual NAS species have been conducted, but these studies have used various and often suboptimal genotyping techniques (Vanderhaeghen et al., 2015). Current understanding regarding evolutionary relationships of NAS and other staphylococcal species is primarily based on information obtained from phylogenetic trees of 16S rRNA gene and various other individual gene/protein sequences (Takahashi et al., 1999; Drancourt and Raoult, 2002; Kwok and Chow, 2003; Shah et al., 2007; Ghebremedhin et al., 2008), which often produces conflicting phylogenies (Ghebremedhin et al., 2008; Lamers et al., 2012). Secondly, most previous studies attempted to resolve the *Staphylococcus* phylogeny from a variety of hosts, including humans and animals. Unfortunately, a reliable hierarchical classification of NAS species of bovine origin, which reveals true evolutionary histories and speciation, has not yet been reported.

In this study, WGS-based phylogenetic trees, constructed using ML, MP, and NJ methods, employing different evolutionary models, divided bovine NAS species into five distinct monophyletic clades. Earlier studies based on single genes and concatenated sequences suggested from 4 to 11 species groups in staphylococci (Kloos et al., 1998a; Takahashi et al., 1999; Drancourt and Raoult, 2002; Kwok and Chow, 2003; Shah et al., 2007; Ghebremedhin et al., 2008; Lamers et al., 2012). Variability in these groupings was attributed to input data sets, methods, and evolutionary models of tree construction.

WGS-based phylogenies of bovine NAS, constructed in this study, produced consensus grouping, and interspecies relationships when tested against various methods (ML, MP, and NJ) and evolutionary models [Jones-Taylor-Thornton (JTT), Whelan and Goldman (WAG), and Subtree-Pruning-Regrafting (SPR) models]. There were similar major clades in our multilocus trees (ML and NJ) when tested against General Time Reversible, Subtree-Pruning-Regrafting, and Kimura 2-parameter models. However, there were variable relationships of the species within larger clades in these trees. Division of bovine NAS into five major clades represent ancient events in evolutionary history of NAS, whereas the branching order within clades (toward the tip of the trees) represent recent evolutionary changes. A recent study on staphylococcal phylogeny, using a multilocus data set of five genes, inferred consistent placement of species near the tips of the trees, but also indicated limitations of inferring phylogenies of higher taxa (Lamers et al., 2012). However, in our WGS-based phylogenies, there was a consistent grouping of larger clades (ancient evolution) and species relationships (recent evolutionary histories) for NAS species obtained in all five WGS trees.

For this study, we also constructed 72 single protein trees (24 ML, 24 MP, and 24 NJ), 15 single gene trees (5 ML, 5 MP, and 5 NJ), and three multilocus trees (ML, MP, and NJ). The purpose of these phylogenetic reconstructions was to observe effects of various data sets, methods, and evolutionary models on inferring phylogenetic relationships among bovine NAS species. Interestingly, none of the single gene/protein trees, constructed using any of the phylogenetic estimation methods, produced identical consensus phylogeny to WGS phylogenetic reconstruction. There were several unresolved, inconsistent, and conflicting relationships among NAS in single genes/proteins trees. Therefore, we inferred that single genes/proteins may not be suitable for identification and phylogeny of NAS species. Conflicting results of single genes/proteins are understandable, as phylogenetic reconstructions based upon single genes/proteins are potentially affected by many parameters. Incomplete lineage sorting, gene gain/loss, gene duplication, presence of paralogs, convergent evolution, and horizontal gene transfers interfere with tree construction (Maddison and Knowles, 2006; Burbrink and Pyron, 2011; Chung and Ané, 2011; Mendes and Hahn, 2016; Stadler et al., 2016). Additionally, each gene may be under different selection pressure and therefore may have been evolving differently, resulting in differing phylogenies (Degnan and Rosenberg, 2006; Burbrink and Pyron, 2011).

In our single proteins phylogenetic estimations of bovine NAS species, Clades A and B were recovered in most reconstructions (Table 6). However, there were more conflicting results for species composition and species relationships in Clades D and E. Therefore, we inferred that that evolutionary interval after speciation may also have affected phylogenetic relationships. For example, in case of Clades A and B of bovine NAS (which represent earliest lineages), sufficient evolutionary changes have accumulated to differentiate respective species of these clades. This is in contrast with Clade D and Clade E, which were more recent lineages that have not resolved properly in most of our single proteins phylogenies, as previously concluded (Takahashi et al., 1999; Kwok and Chow, 2003; Shah et al., 2007; Ghebremedhin et al., 2008). Additionally, it is well-established that single-gene trees reflect evolution of particular genes and do not necessarily relate to speciation. Hence, single gene phylogenies may not be optimal for studying species relationships (Beiko et al., 2005; Puigbò et al., 2010).

All species descending from a common ancestor are expected to share some biochemical, phenotypical or physiological characteristics. In our WGS trees, placement of *S. sciuri, S. fleurettii*, and *S. vitulinus* into Clade A, which represented oxidase-positive and novobiocin-resistant members, often defined as “Sciuri group,” was consistent with previous studies (Drancourt and Raoult, 2002; Kwok and Chow, 2003; Svec et al., 2004; Shah et al., 2007; Lamers et al., 2012). All remaining bovine NAS species were oxidase-negative, indicating that the gene responsible for oxidase activity was lost after divergence of Clade A. Species included in Clade B were oxidase-negative, novobiocin-susceptible, and coagulase variable, including *S. chromogenes* which is coagulase-negative and *S. hyicus* and *S. agnetis* which are coagulase variable. Within this clade, *S. chromogenes* is, with ~50% of NAS isolations from bovine milk samples, the most prevalent species in IMI in Canada (Condas et al., in review) and worldwide (Sampimon et al., 2009; Piessens et al., 2011). Placement of Clade B as second monophyletic lineage of NAS species, supported by 100% nodal support, was unique to our WGS and multilocus trees. Species of this clade was given variable placement and species relatedness in previous reconstructions from multilocus data set (Lamers et al., 2012) and various single genes trees, including, 16S rRNA, *hsp60, rpoB, sodA, gap, tuf*, and *dnaJ* sequences (Takahashi et al., 1999; Drancourt and Raoult, 2002; Kwok and Chow, 2003; Shah et al., 2007; Ghebremedhin et al., 2008). All our WGS trees and multilocus trees had monophyletic branching of Clade B as the second lineage of NAS. However, variable phylogenetic placement of this clade was observed in single genes/proteins trees.

Clade C appeared as third lineage in our WGS trees and is composed of multiple isolates of *S. simulans*, the second most prevalent NAS species found in bovine IMI in Canada (Condas et al., in review). These isolates are novobiocin-susceptible, coagulase and oxidase-negative in phenotype. Our placement of *S. simulans* as third lineage was consistent with earlier studies (Lamers et al., 2012). However, discrepancy in branching of this clade was noted in many of our single-protein phylogenies (Tables 3–6) and in previous single-gene phylogenetic reconstructions (Takahashi et al., 1999; Kwok and Chow, 2003; Ghebremedhin et al., 2008). Species included in Clade D of our WGS trees, were novobiocin-susceptible, oxidase-negative, and coagulase-negative. The species of this clade were further divided into three cluster groups, consistent with a previous classification (Lamers et al., 2012). Species included in Clade E were novobiocin-resistant, oxidase-negative and coagulase-negative, except *S. auricularis* which was novobiocin susceptible, oxidase-negative and coagulase-negative (Lamers et al., 2012). Our placement of *S. auricularis* sharing a common ancestor with the other species of Clade E, was generally in agreement with previous 16S rRNA gene based phylogenies (Takahashi et al., 1999; Ghebremedhin et al., 2008) but contradictory to another phylogeny (Lamers et al., 2012) that proposed *S. auricularis* as a second divergence group within Staphylococci. However, there was a different pattern of branching for *S. auricularis* in their 16S rRNA and *dnaJ* base phylogenetic reconstructions (Lamers et al., 2012). Our placement of *S. auricularis* with other species of Clade E was consistent in all WGS and multilocus trees. Perhaps differences between current and previous (Lamers et al., 2012) multilocus reconstructions were due to our analyses restricted to NAS of bovine origin, and excluded NAS species from other hosts. Notwithstanding, WGS data enabled us to construct robust phylogeny of bovine NSA isolates obtained from CBMQRN. Based on these results, an association can now be investigated between phylogeny and epidemiology of these isolates. This study represents the first multi-genome-scale phylogenetic analysis of bovine NAS isolates.

## Conclusions

Based on WGS, we established a robust phylogeny of bovine NAS species, supported by various methods and models of evolutionary estimations. Evolutionary and interspecies relationships revealed by this phylogeny will allow determination, among related species, of common biological traits, such as virulence, environmental niche, and geographical distribution, and host specificity. Knowing the true phylogeny of NAS species will also provide the basis for studies to elucidate the role and significance of individual and related NAS species in udder health.

We also compared WGS phylogenetic trees with single genes/proteins and multilocus trees. Based on our results, we conclude that whole genome sequences, which encompass the entire genetic information of organisms, offer great accuracy in reconstructing evolutionary relationships and should be “the method of choice” for identification and elucidation of evolutionary histories of bacterial organisms. Inferences derived from this work will be helpful in selection of parameters, such as input data, evolutionary models and methods of phylogenetic reconstruction for many other groups of bacteria.

## Author contributions

Conceived and designed the experiments: SN, HB, CL, and JD. Performed lab experiments: SN, LC, DN, and DC. Computational analyses and analyzed the data: SN and JD. Wrote the paper: SN, HB, and JD.

### Conflict of interest statement

The authors declare that the research was conducted in the absence of any commercial or financial relationships that could be construed as a potential conflict of interest.
