# Supplementary material for: Comprehensive Phylogenetic Analysis of Bovine Non-aureus Staphylococci Species Based on Whole-Genome Sequencing
Source: Front Microbiol. 2016 Dec 20;7:1990. doi: 10.3389/fmicb.2016.01990 (PMC5168469; doi:10.3389/fmicb.2016.01990)
Supplement: Supplementary file 1 [file Table1.pdf]

Sup. Table 1: List of conserved proteins used for phylogenetic analyses

1. CDP diglyceride synthetase (CdsA)
2. Elongation factor G (EF-G)
3. Elongation factor Tu (EF-Tu)
4. Elongation factor P (EF-P)
5. Cell Division protein FtsY
6. Serine hydroxymethyl transferase, GlyA
7. Gyrase A (GyrA)
8. Gyrase B (GyrB)
9. Hsp60
10. Hsp70
11. Dimethyladenosine transferase, KsgA
12. RecA
13. Ribosomal protein L1
14. Ribosomal protein L2
15. Ribosomal protein S15
16. Ribosomal protein S2
17. Ribosomal protein S8
18. Ribosomal protein S11
19. RNA polymerase α subunit (RpoA)
20. RNA polymerase β subunit (RpoB)
21. RNA polymerase β' subunit (RpoC)
22. Preprotein translocase SecA
23. Preprotein translocase SecY
24. DNA helicase (PcrA)
