# Supplementary material for: Comprehensive Phylogenetic Analysis of Bovine Non-aureus Staphylococci Species Based on Whole-Genome Sequencing
Source: Front Microbiol. 2016 Dec 20;7:1990. doi: 10.3389/fmicb.2016.01990 (PMC5168469; doi:10.3389/fmicb.2016.01990)
Supplement: Supplementary file 2 [file SupplementaryFigures1-20.pdf]

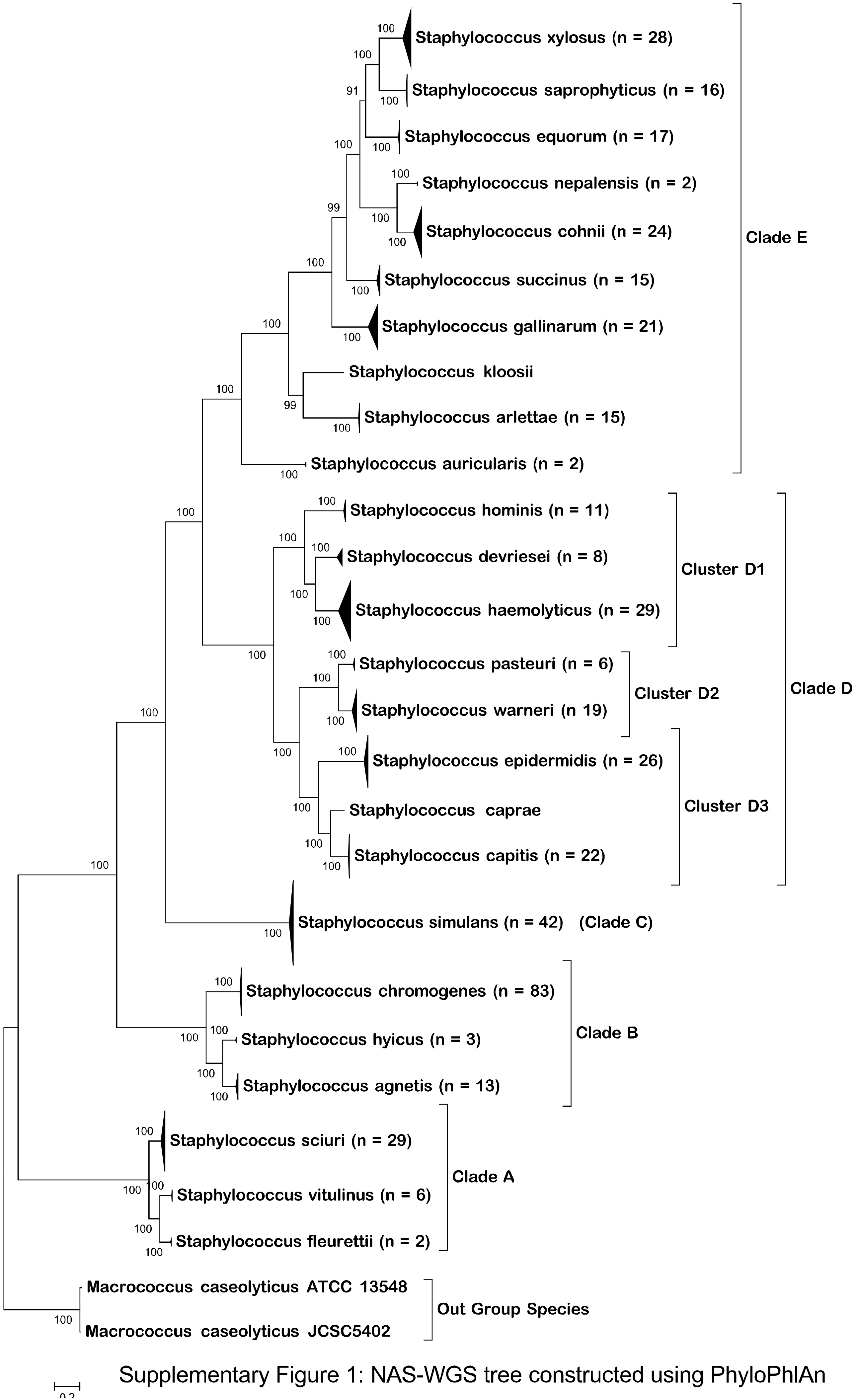

Supplementary Figure 1: NAS-WGS tree constructed using PhyloPhlAn

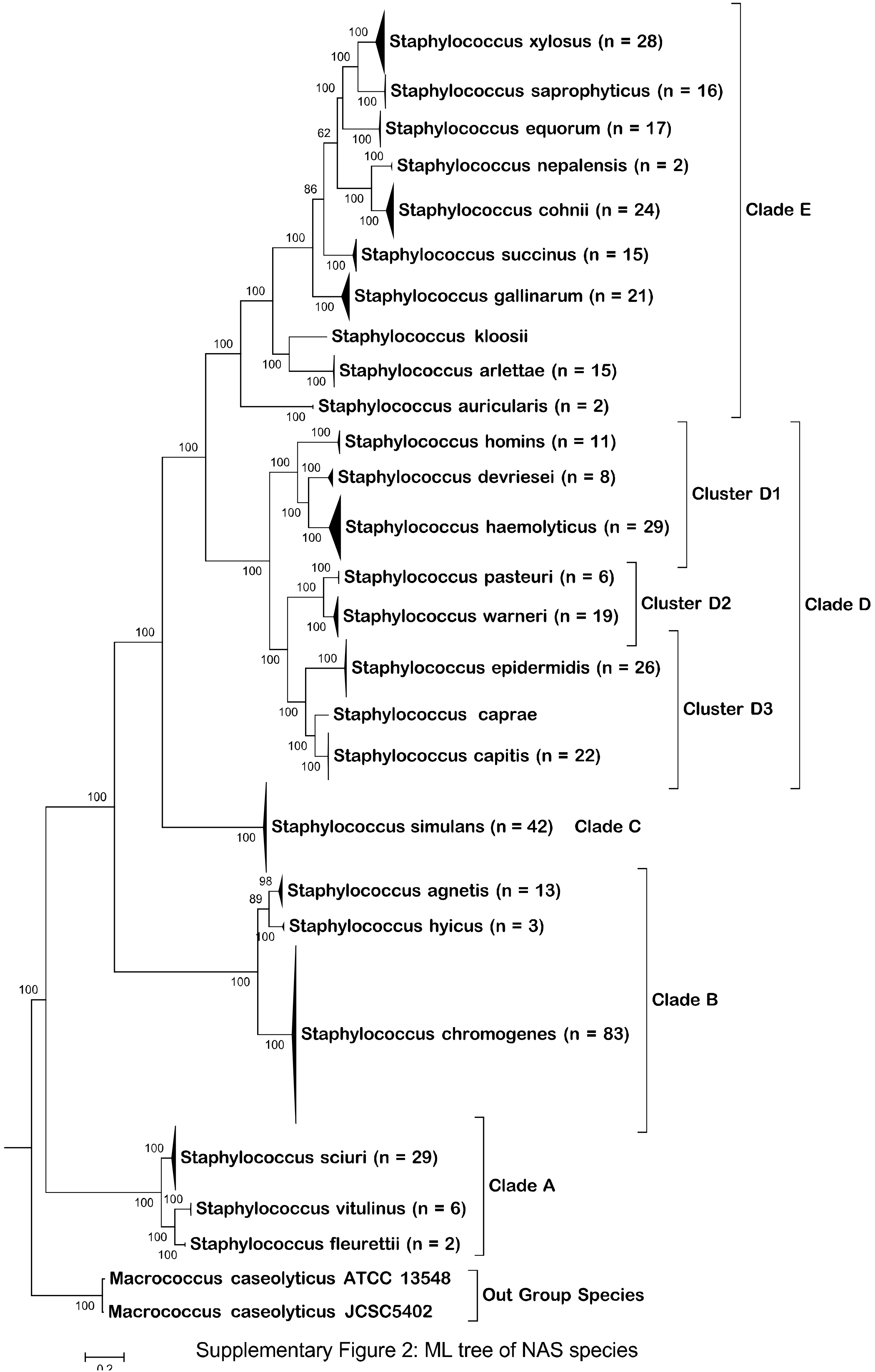

Supplementary Figure 2: ML tree of NAS species

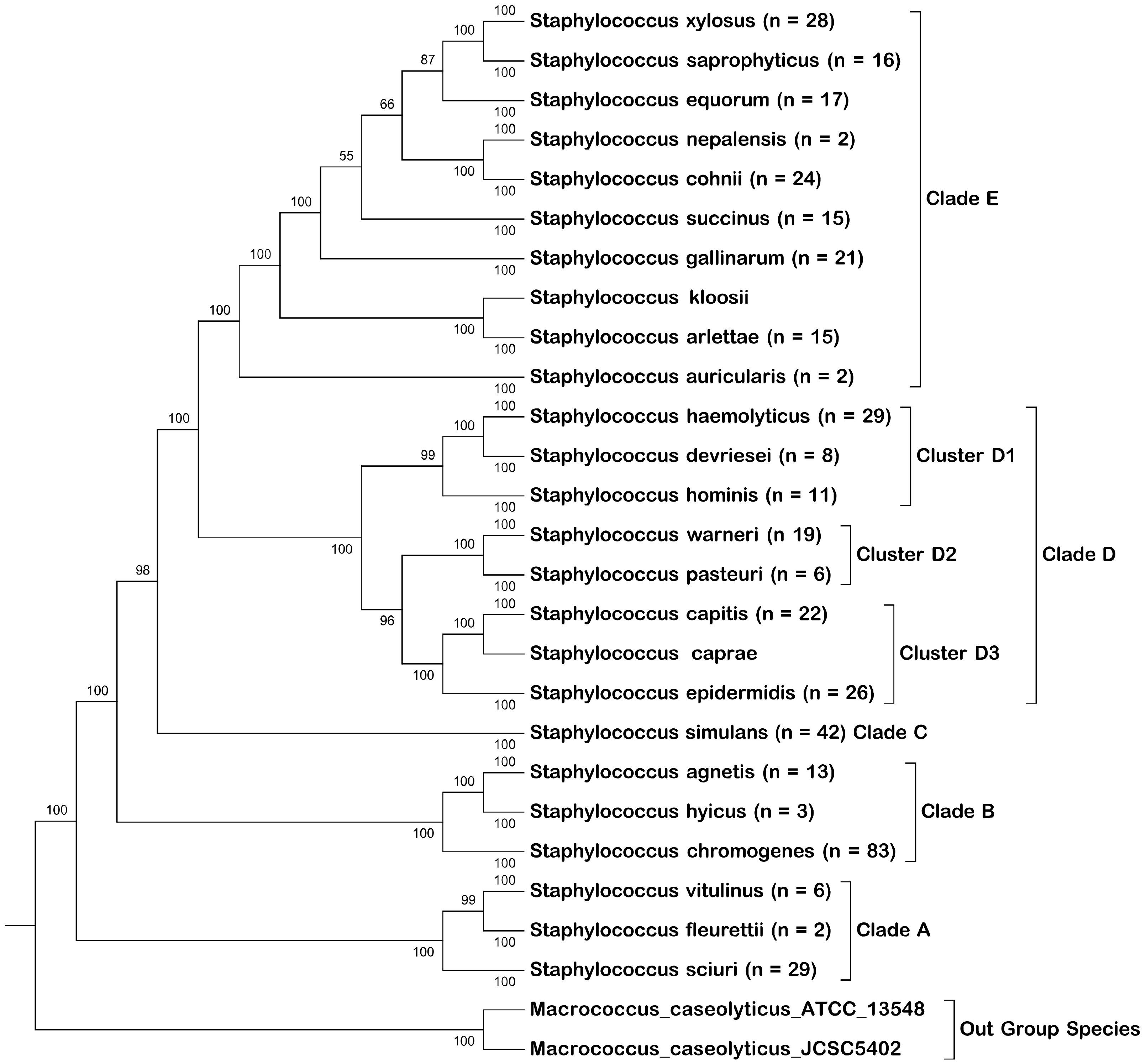

Supplementary Figure 3: ML tree of NAS species

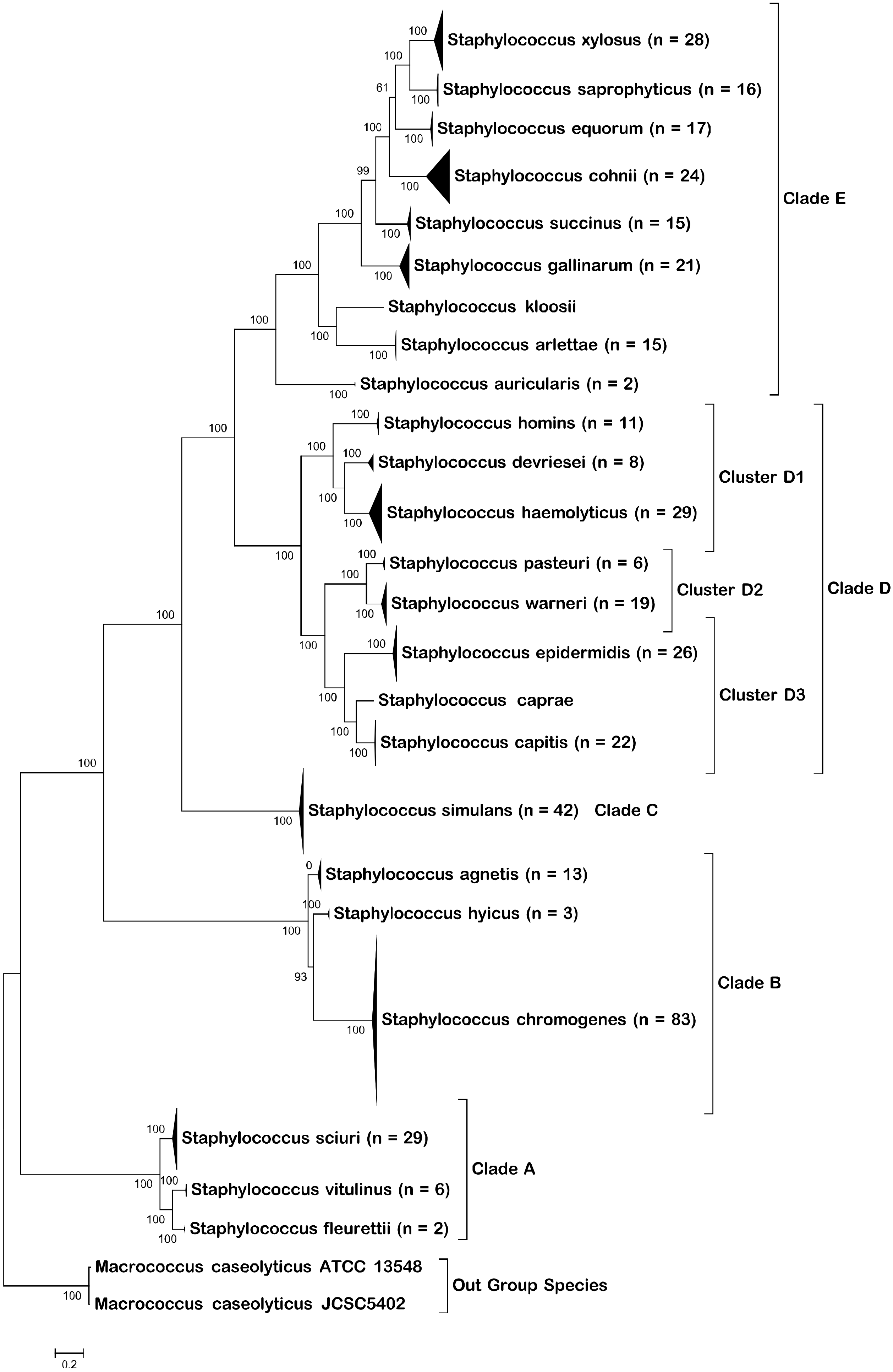

Supplementary Figure 4: NJ tree of NAS species

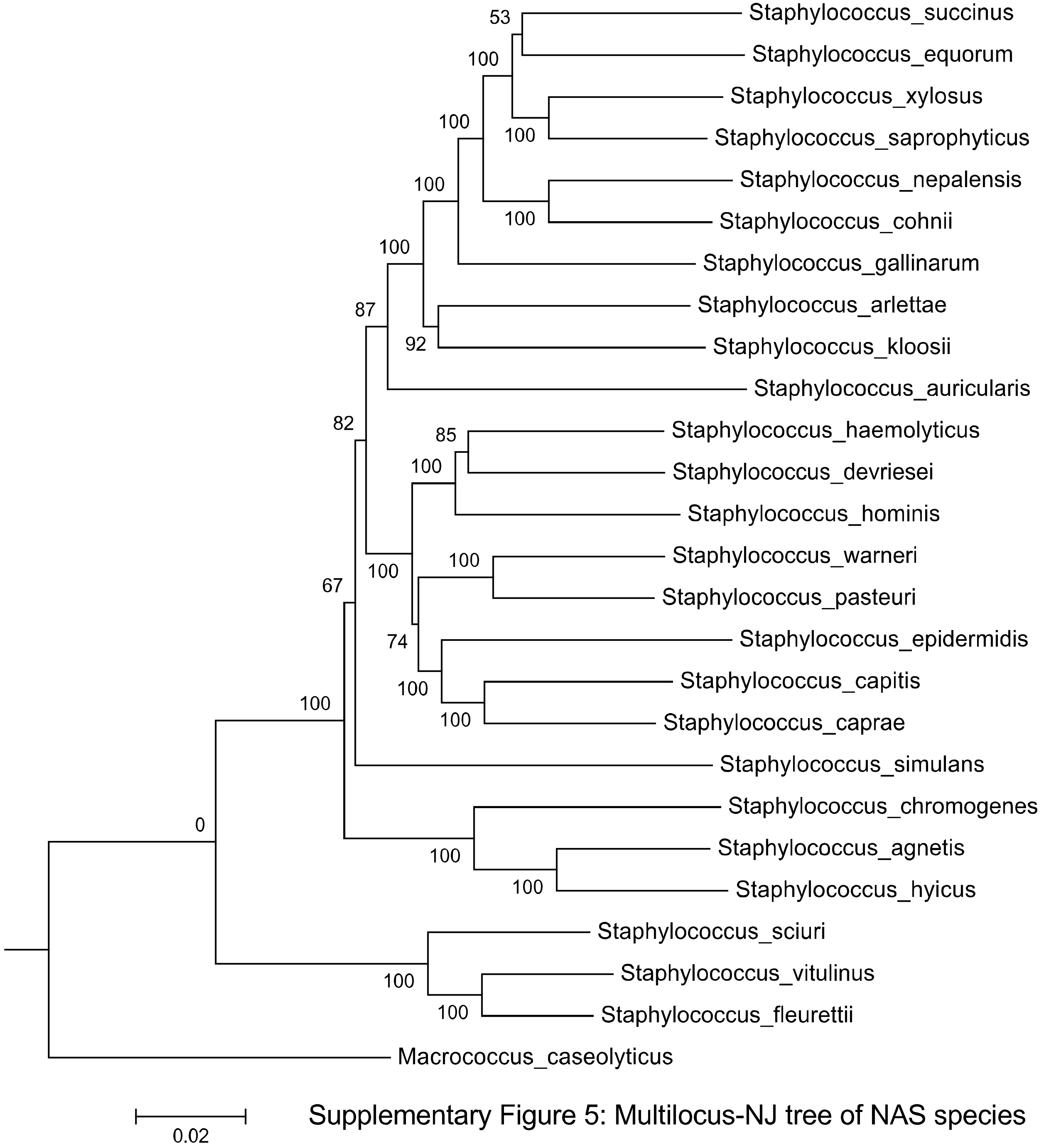

Supplementary Figure 5: Multilocus-NJ tree of NAS species

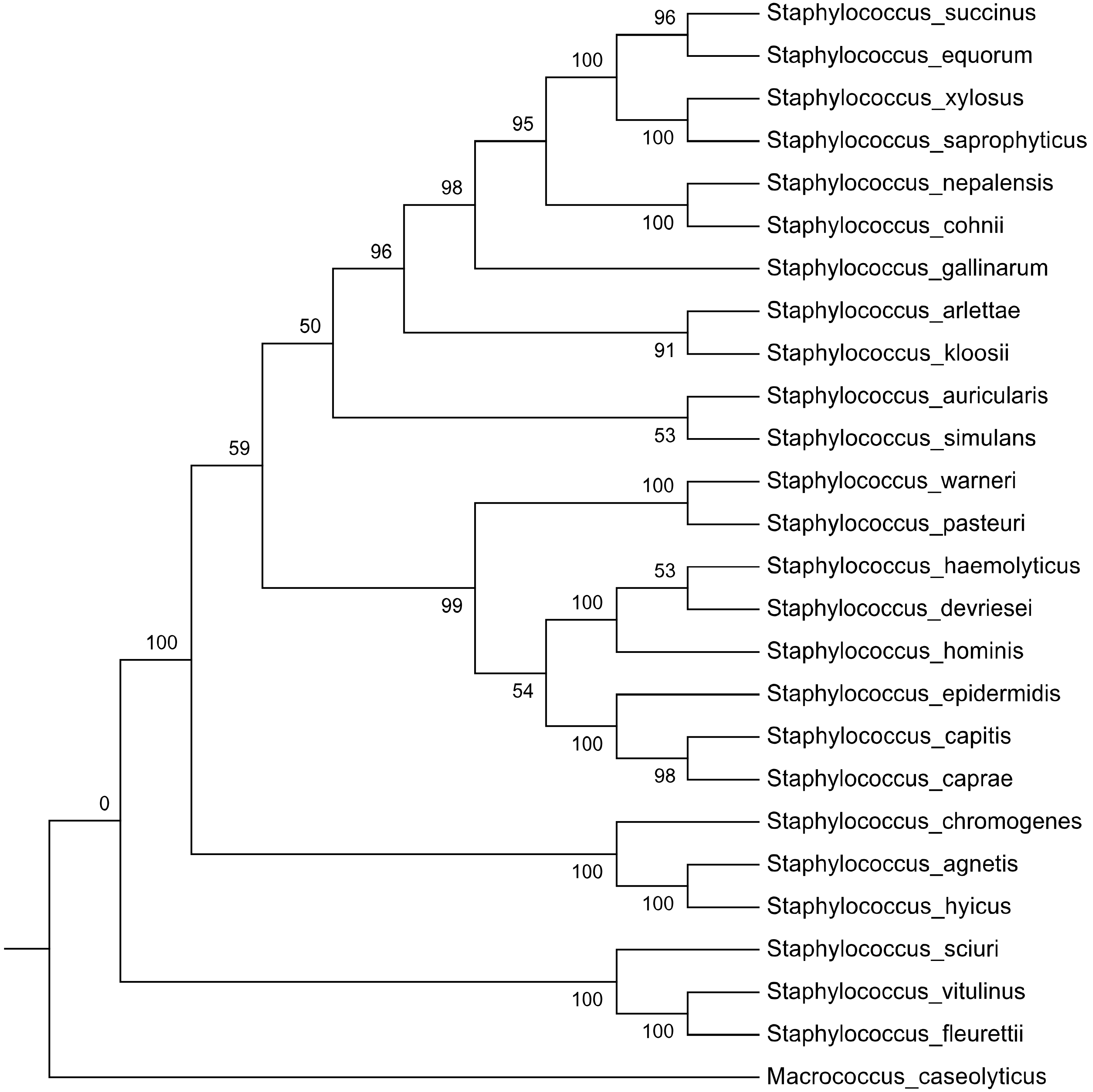

Supplementary Figure 6: Multilocus-MP tree of NAS species

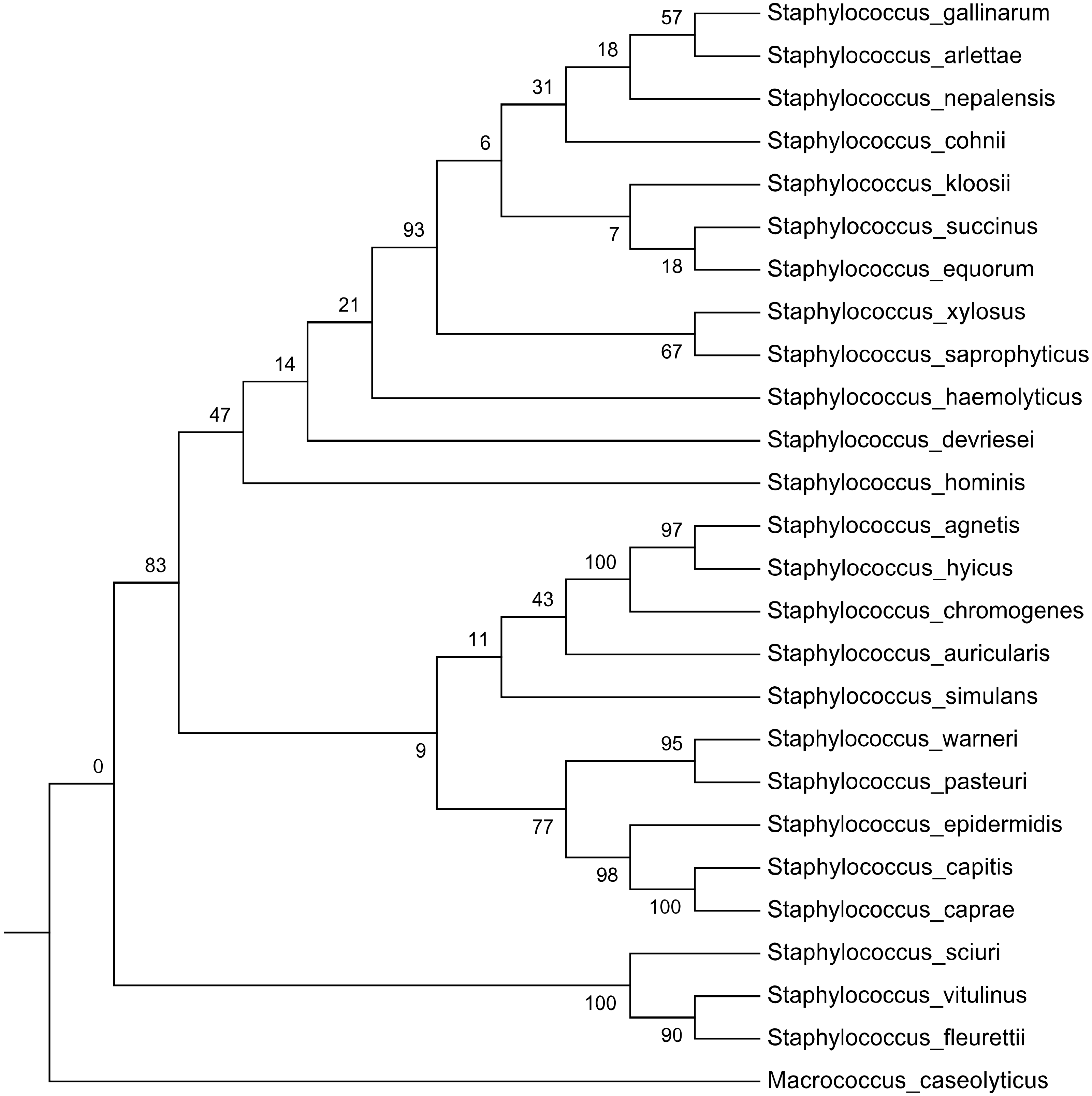

Supplementary Figure 7: 16S rRNA-MP tree of NAS species

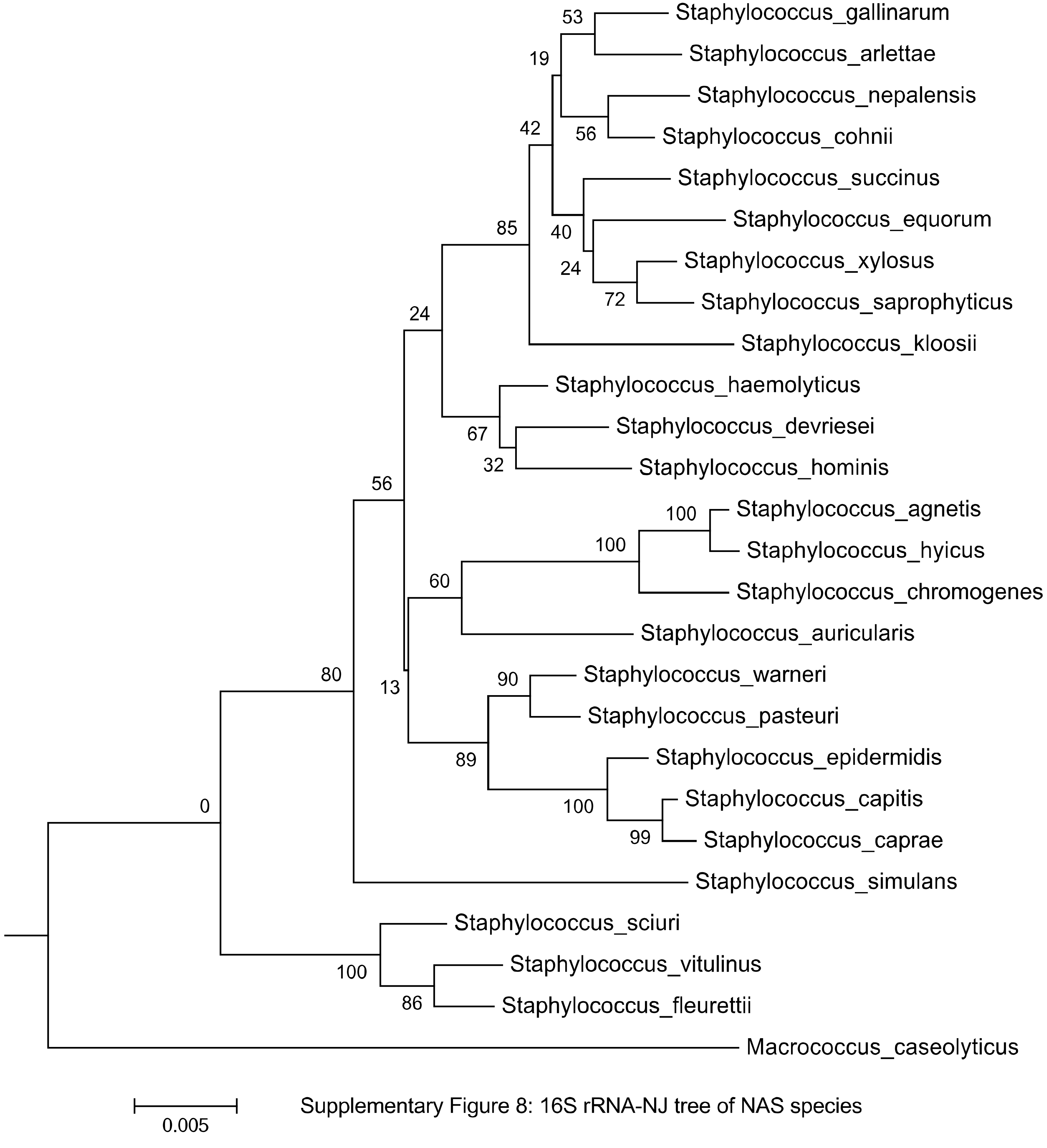

Supplementary Figure 8: 16S rRNA-NJ tree of NAS species

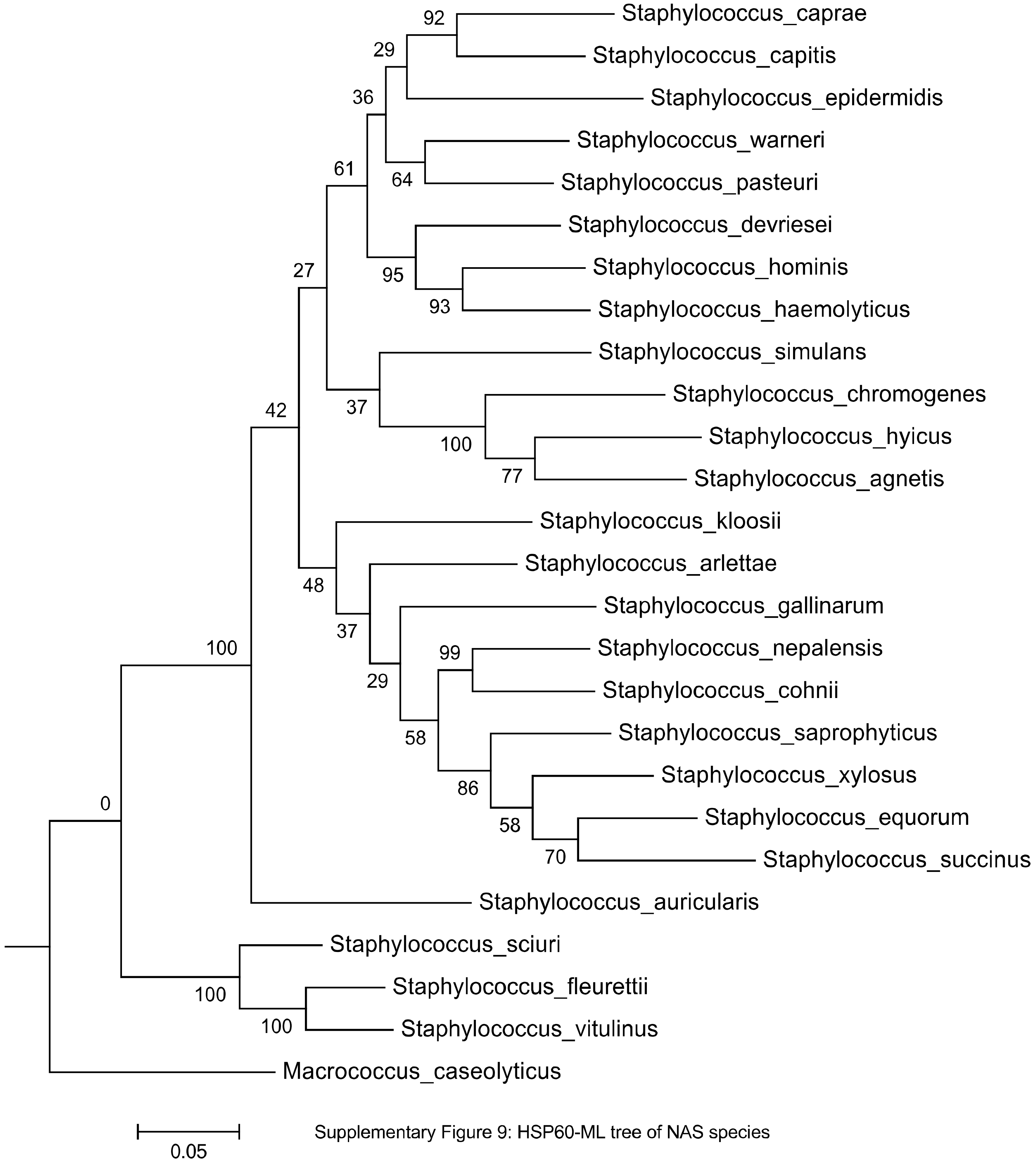

Supplementary Figure 9: HSP60-ML tree of NAS species

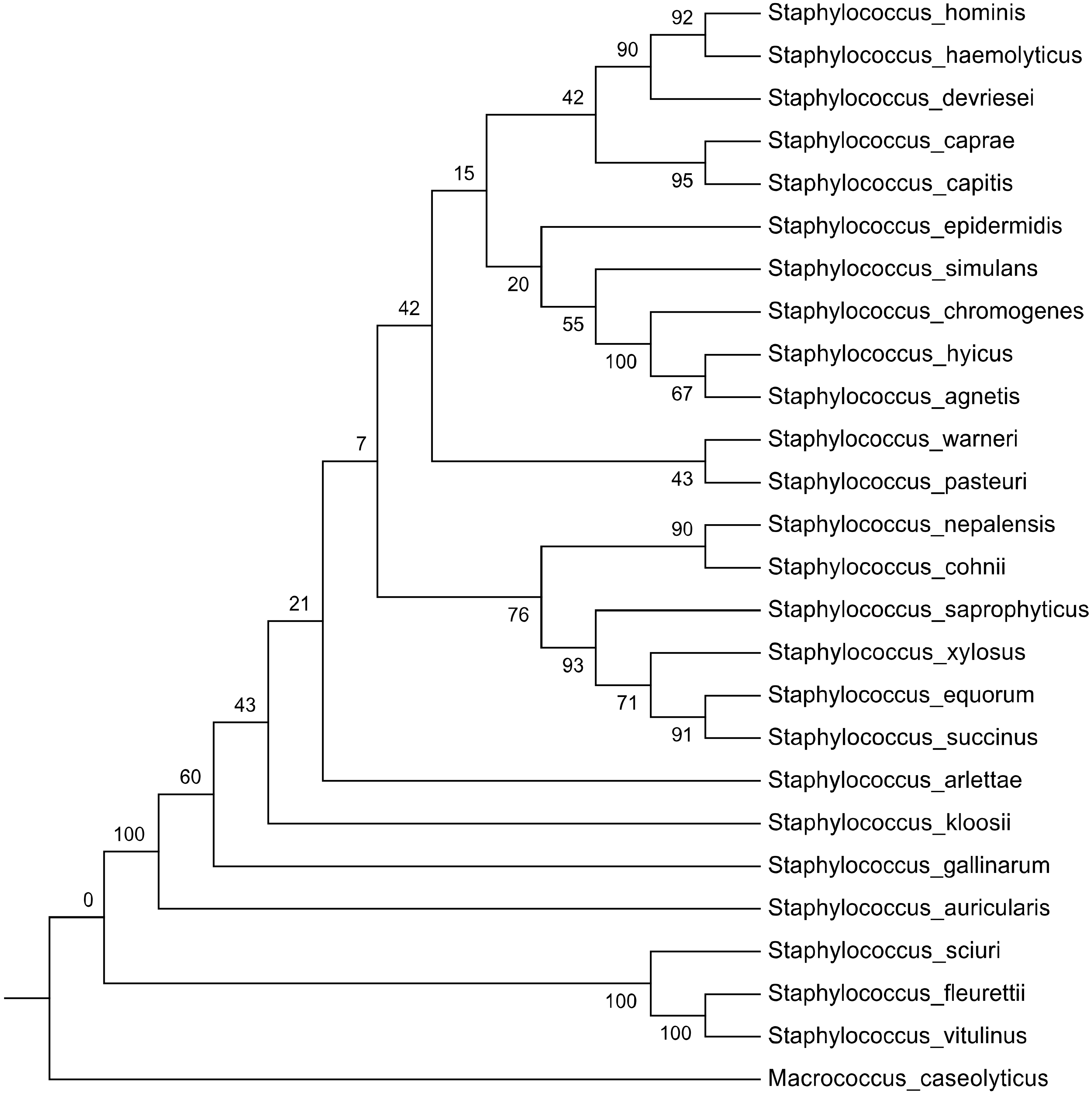

Supplementary Figure 10: HSP60-MP tree of NAS species

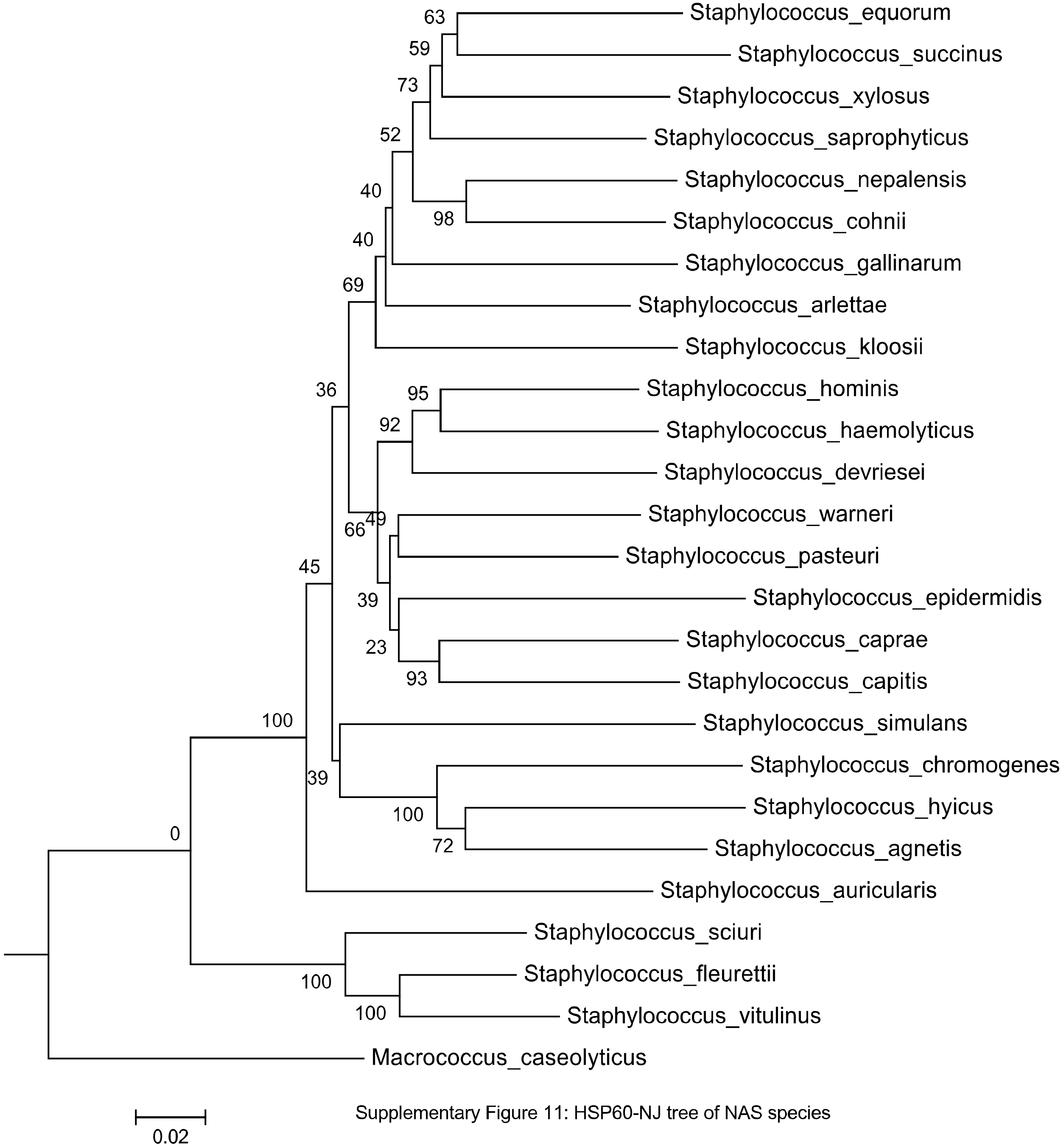

Supplementary Figure 11: HSP60-NJ tree of NAS species

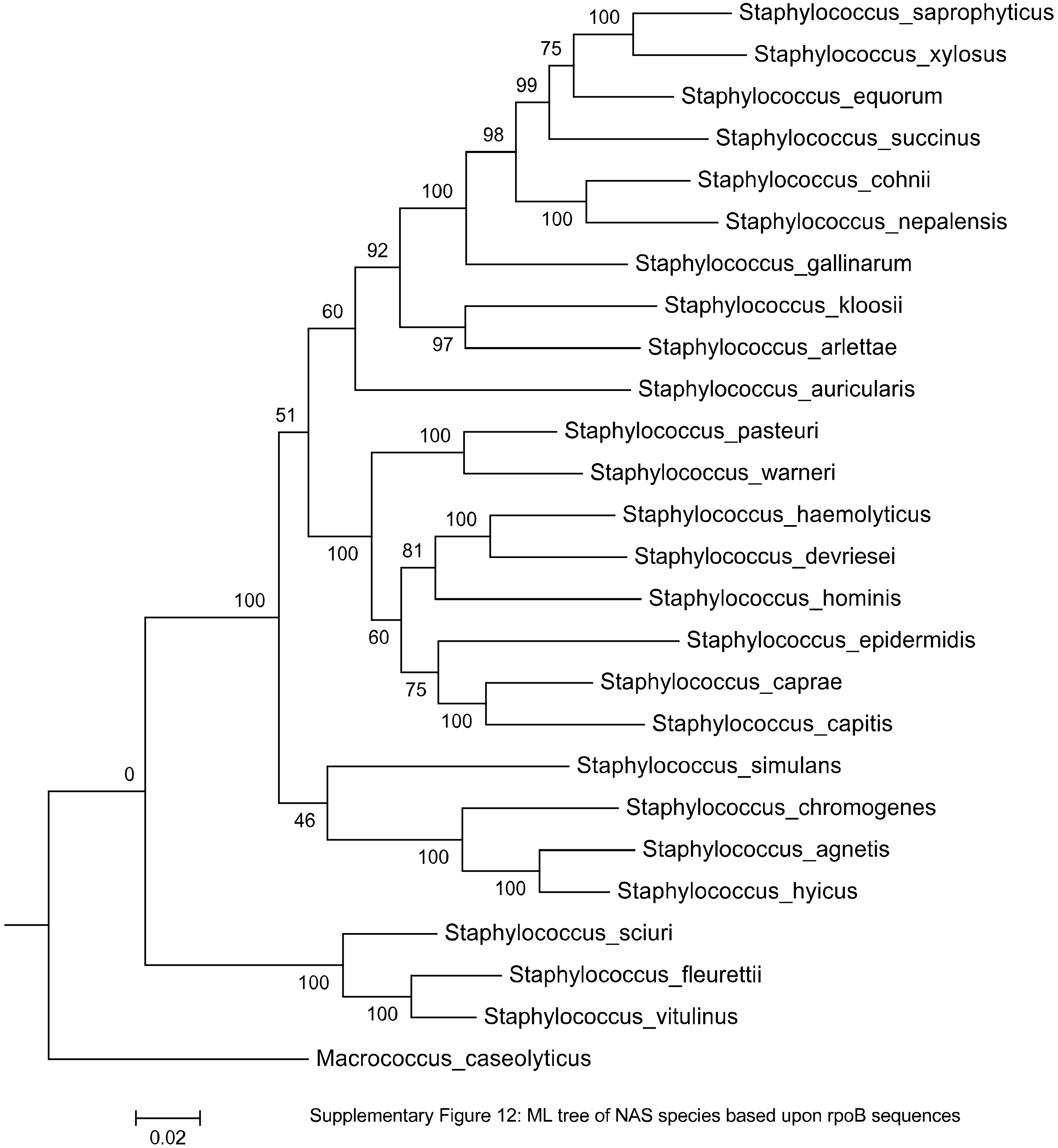

Supplementary Figure 12: ML tree of NAS species based upon rpoB sequences

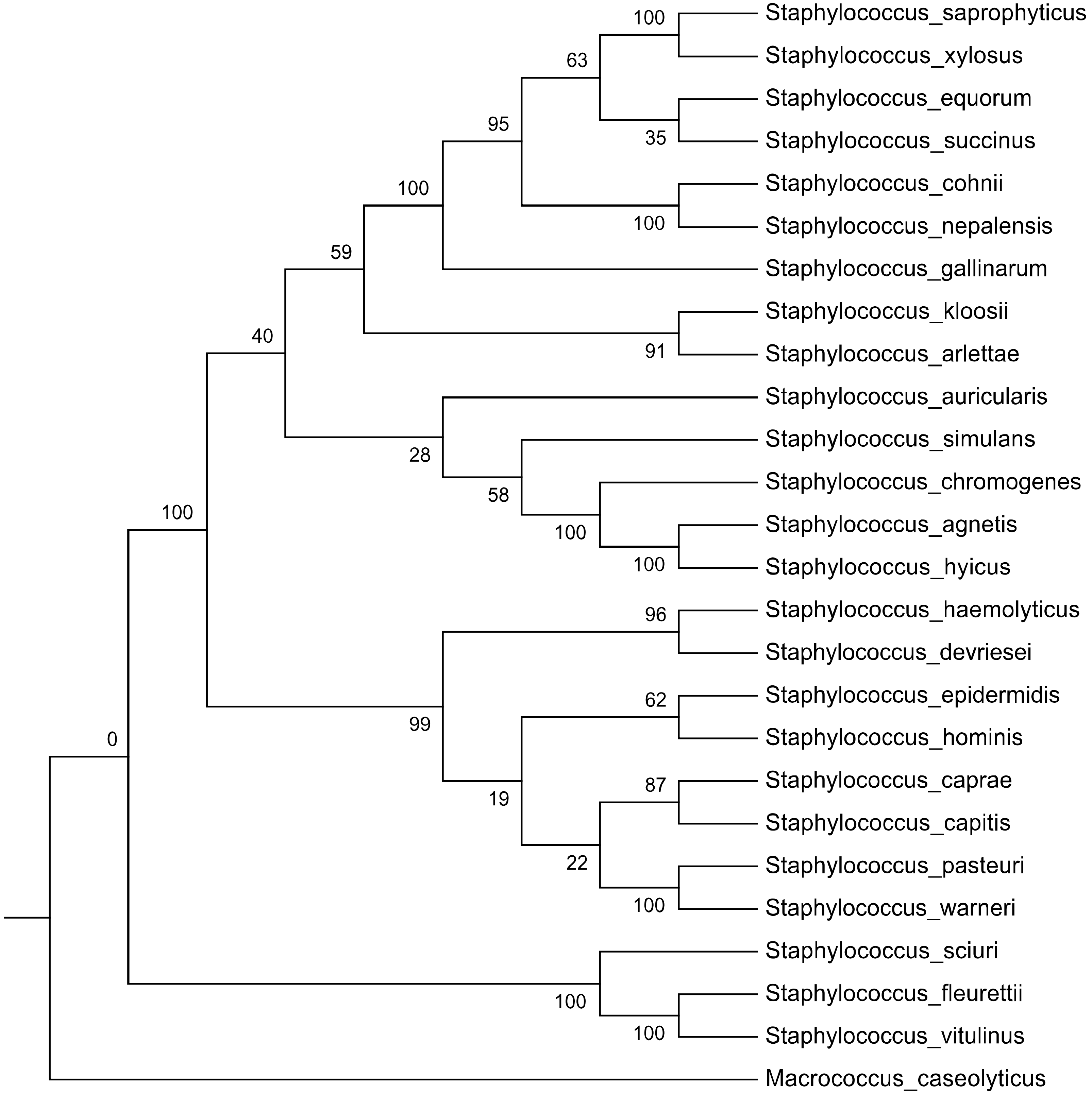

Supplementary Figure 13: MP tree of NAS species based upon rpoB sequences

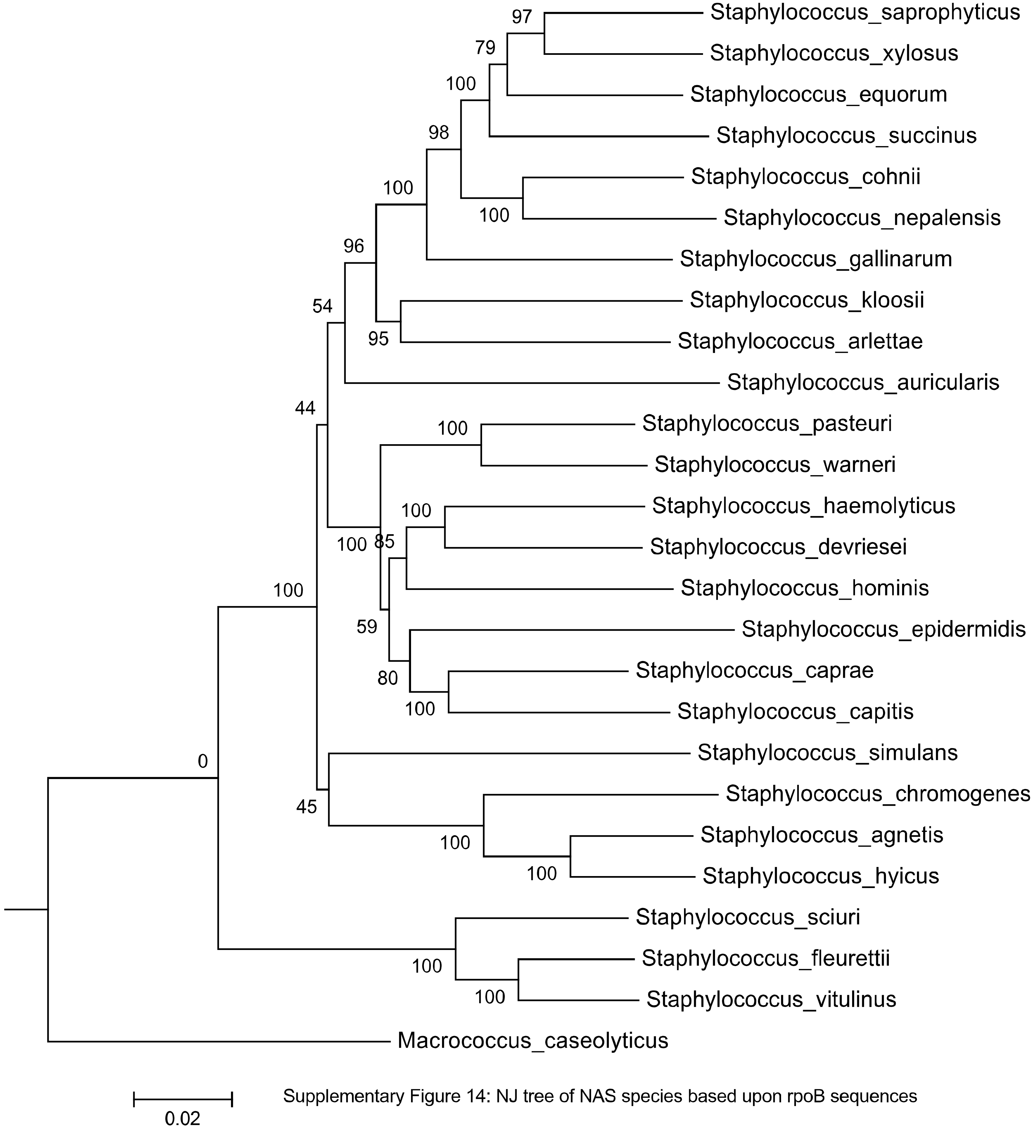

Supplementary Figure 14: NJ tree of NAS species based upon rpoB sequences

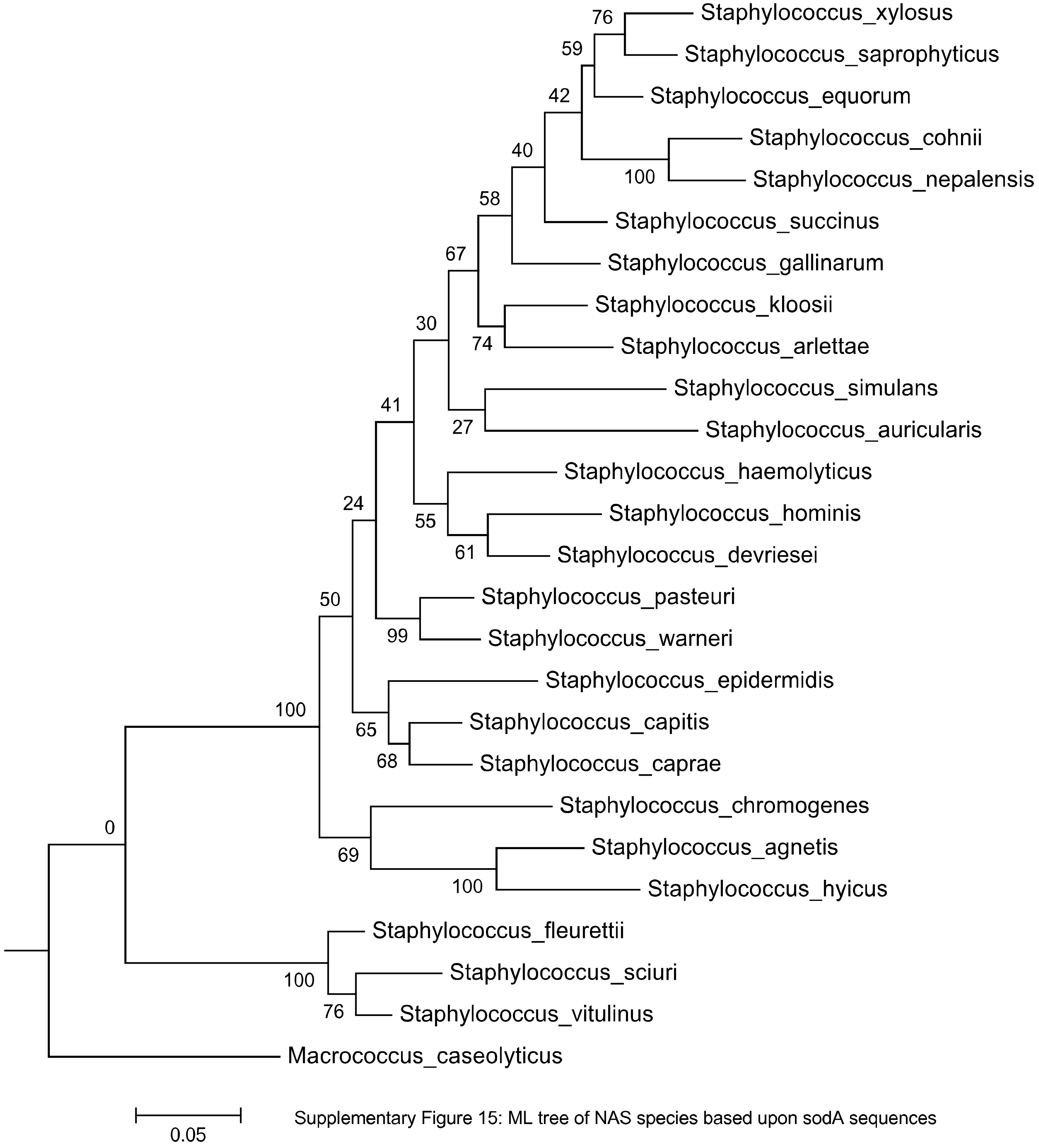

Supplementary Figure 15: ML tree of NAS species based upon sodA sequences

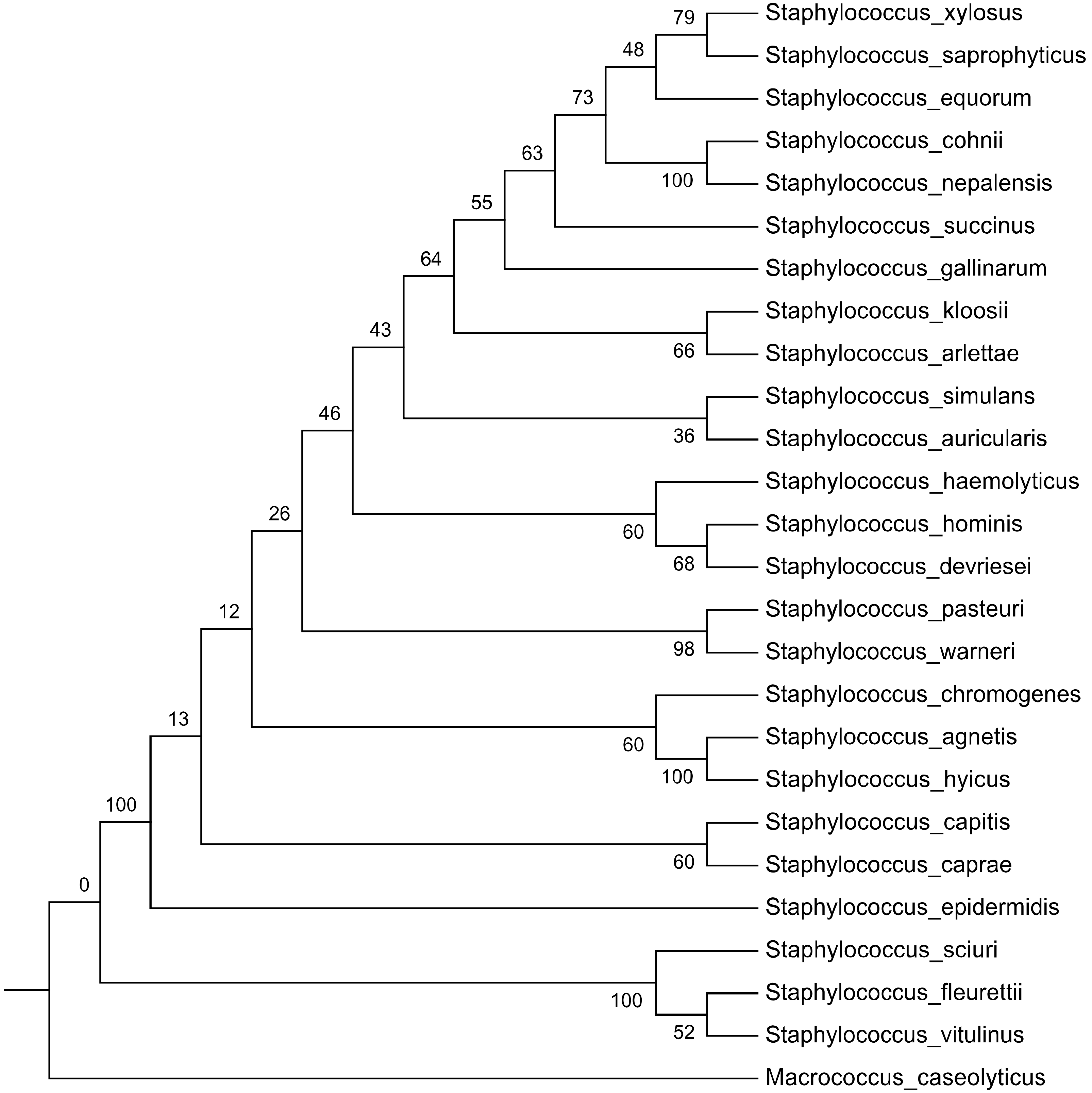

Supplementary Figure 16: MP tree of NAS species based upon sodA sequences

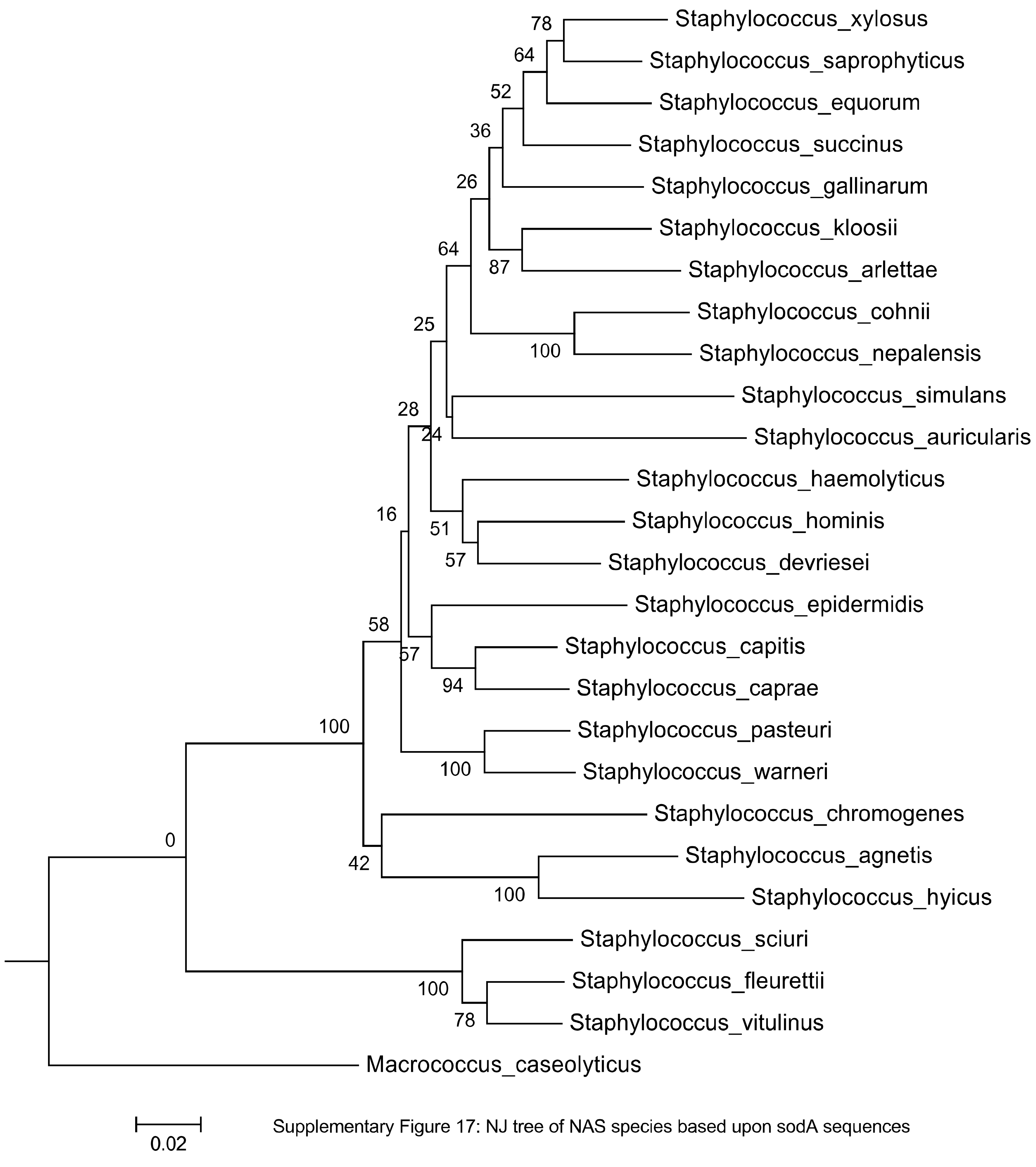

Supplementary Figure 17: NJ tree of NAS species based upon sodA sequences

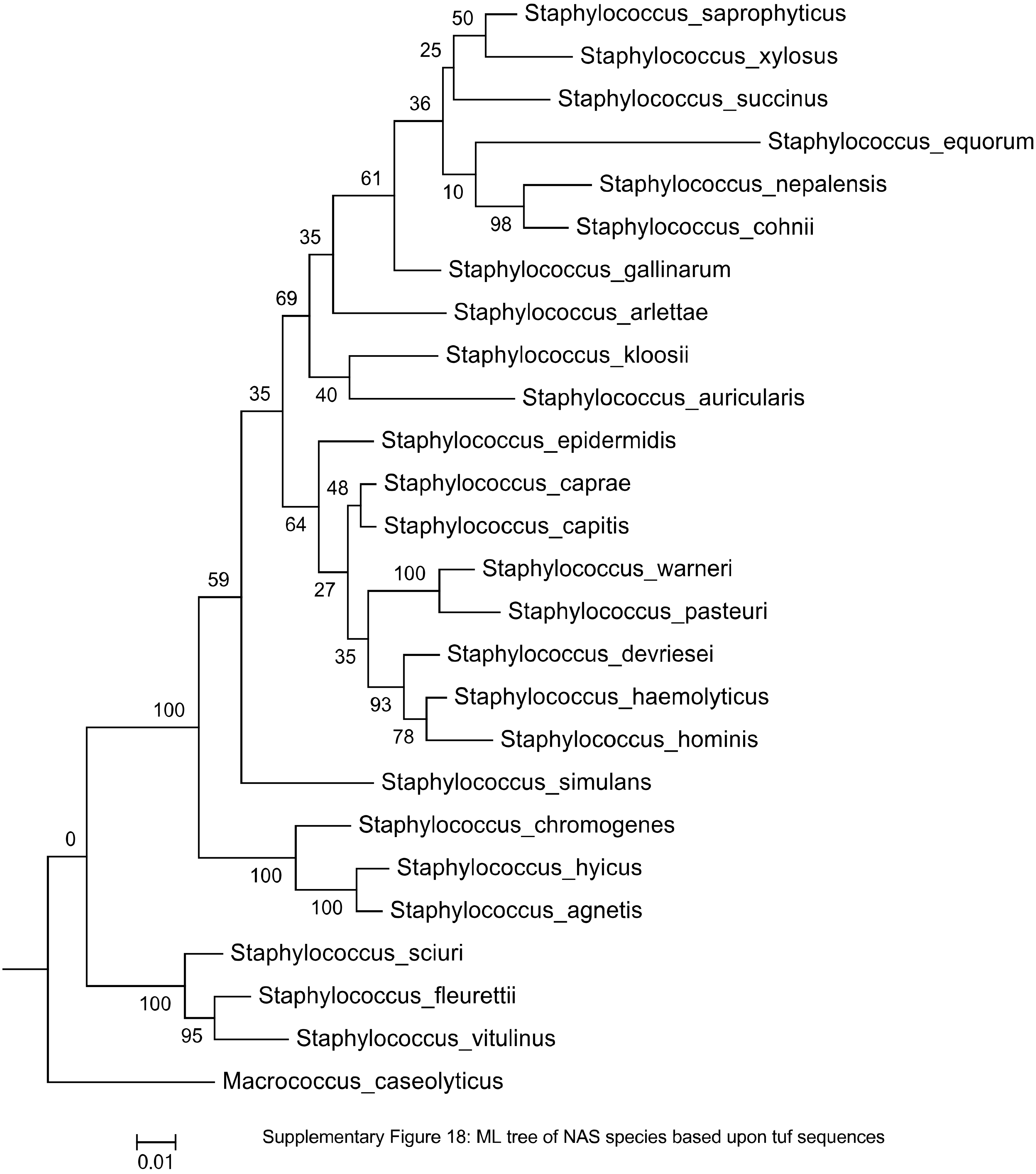

Supplementary Figure 18: ML tree of NAS species based upon tuf sequences

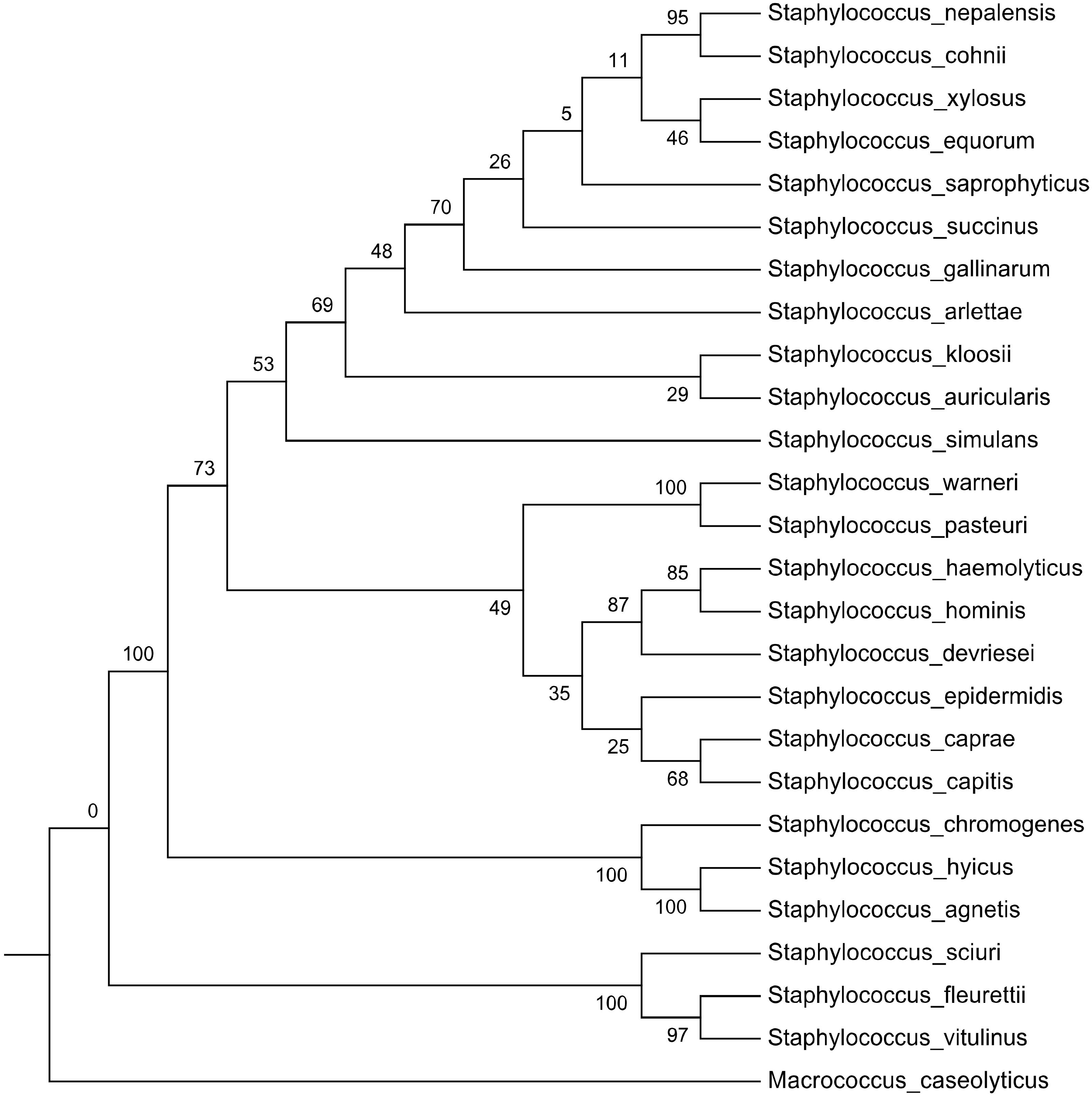

Supplementary Figure 19: MP tree of NAS species based upon tuf sequences

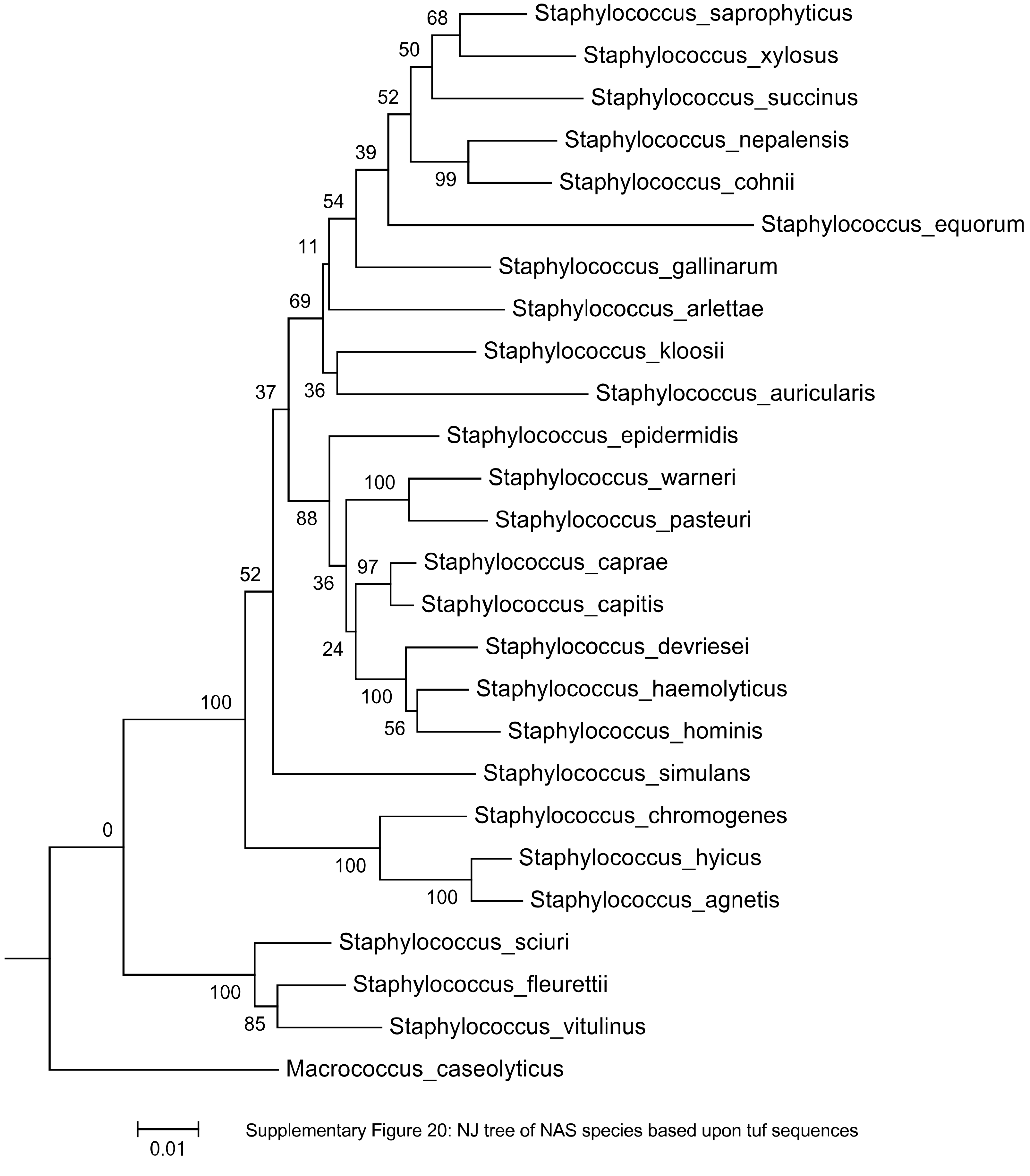

Supplementary Figure 20: NJ tree of NAS species based upon tuf sequences
